# Vascular Endothelial Growth Factor in Cartilage Development and Osteoarthritis

**DOI:** 10.1038/s41598-017-13417-w

**Published:** 2017-10-12

**Authors:** Masashi Nagao, John L. Hamilton, Ranjan Kc, Agnes D. Berendsen, Xuchen Duan, Chan Wook Cheong, Xin Li, Hee-Jeong Im, Bjorn R. Olsen

**Affiliations:** 1 Department of Developmental Biology, Harvard School of Dental Medicine, 188 Longwood Avenue, Boston, MA 02115 USA; 2 Department of Biochemistry, Rush University Medical Center, 1735 W, Harrison Street, Chicago, IL 60612 USA; 3 Jesse Brown Veterans Affairs (VA) Medical Center, 820S, Damen Avenue, Chicago, IL 60612 USA; 4 Department of Bioengineering, University of Illinois, Chicago, IL 60612 USA; 5 Department of Orthopaedic Surgery, Juntendo University School of Medicine 2-1-1 Hongo Bunkyo-ku, Tokyo, 113-8421 Japan

## Abstract

Genome wide studies indicate that vascular endothelial growth factor A (VEGF) is associated with osteoarthritis (OA), and increased VEGF expression correlates with increased disease severity. VEGF is also a chondrocyte survival factor during development and essential for bone formation, skeletal growth and postnatal homeostasis. This raises questions of how the important embryonic and postnatal functions of VEGF can be reconciled with an apparently destructive role in OA. Addressing these questions, we find that VEGF acts as a survival factor in growth plate chondrocytes during development but only up until a few weeks after birth in mice. It is also required for postnatal differentiation of articular chondrocytes and the timely ossification of bones in joint regions. In surgically induced knee OA in mice, a model of post-traumatic OA in humans, increased expression of VEGF is associated with catabolic processes in chondrocytes and synovial cells. Conditional knock-down of *Vegf* attenuates induced OA. Intra-articular anti-VEGF antibodies suppress OA progression, reduce levels of phosphorylated VEGFR2 in articular chondrocytes and synovial cells and reduce levels of phosphorylated VEGFR1 in dorsal root ganglia. Finally, oral administration of the VEGFR2 kinase inhibitor Vandetanib attenuates OA progression.

## Introduction

Osteoarthritis (OA), the most common form of arthritis, is a leading cause of pain and disability. Prevalence and incidence of the disorder are predicted to increase as a result of increased lifespan and obesity^1^. No disease-modifying drug is currently available and no major drug target has been identified^2^.

A recent meta-analysis of nine genome-wide association studies concluded that variations in *vascular endothelial growth factor A* (*VEGF*) and collagen *COL11A1* genes are significantly correlated with OA, in addition to the OA susceptibility locus *GDF5* ^3^. Several studies indicate that increased *VEGF* expression is associated with increased OA severity^4,5^. Injection of VEGF into animal joints induces OA and VEGF stimulates degeneration of articular chondrocytes^6^. Anti-VEGF therapy is therefore emerging as a potential OA treatment^7^.

VEGF is an important mediator of endochondral ossification; an essential process of skeletal development and growth^8^. VEGF also functions as survival factor for growth plate chondrocytes during embryonic development^9,10^. These findings raise questions regarding distinct VEGF mechanisms in chondrocytes during development, postnatal growth and progression of pathological conditions such as OA. To address these questions, we targeted *Vegf* expression in *Col2*-expressing and endothelial lineage cells in mice. In addition, we surgically induced knee OA in a mouse model of traumatic knee OA in humans, and compared the effects of treatments that targeted VEGF and the VEGF receptor 2 (VEGFR2) kinase activity. The data provide novel insights into roles of VEGF in cartilage development and OA progression.

## Results

### Lack of VEGF in *Col2-Cre* lineage cells impairs skeletal growth

**Figure 1:**
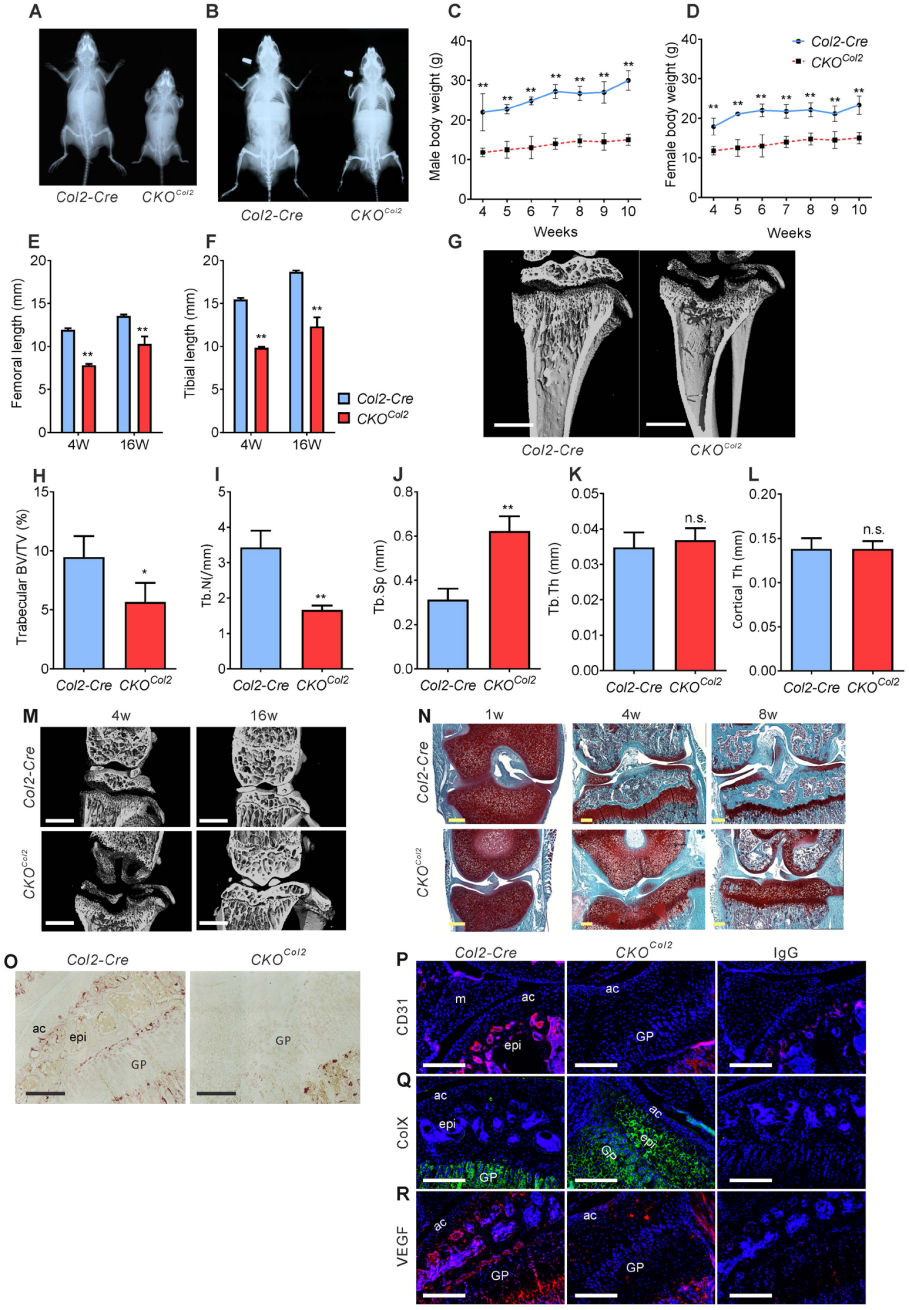
Lack of *Col2-Cre* lineage cell-derived VEGF impairs skeletal growth. X-ray images from 4 (**A**) or 16 (**B**) weeks old *Col2-Cre* and *Col2-Cre;Vegf* ^*fl/fl*^ (*CKO* ^*Col2*^) mice. Reduced body weights in male (**C**) and female (**D**) *CKO* ^*Col2*^ mice from 4 weeks to 10 weeks of age (*n* = 4–10). Reduced femoral (**E**) and tibial (**F**) lengths at 4 and 16 weeks in mutants (*n* = 3–5). Micro-CT images of tibia at 4 weeks (**G**); Scale bars, 1mm. Reduced trabecular bone volume/tissue volume (BV/TV) (**H**), reduced trabecular number (Tb.N) (**I**), and increased trabecular spacing (Tb.Sp) (**J**), but no change in trabecular thickness (Tb.Th) (**K**) and cortical thickness (Cortical Th) (**L**) in *CKO* ^*Col2*^ compared to *Col2-Cre* mice (*n* = 4–5). Micro-CT images of knee joint of 4 and 16 weeks old *Col2-Cre* and C*KO* ^*Col2*^ mice; scale bar, 1mm (**M**). Sections of the knee joint from 1, 4 and 8 weeks (w) old mice stained with Safranin O-fast green (**N**). Sections of tibial epiphysis from 4 weeks old mice stained for Tartrate-resistant Acid Phosphatase (TRAP) (**O**), showing growth plate (GP), articular cartilage (ac) and epiphyseal (epi) regions. Immunofluorescence of articular cartilage (ac), meniscus (m), epiphyseal (epi) and growth plate (GP) regions for CD31 (**P**), collagen X (**Q**) and VEGF (**R**); scale bars, 250 μm; DAPI (blue) for nuclear staining. Sections of *Col2-Cre* mice were used for control antibody (IgG) staining. Data represent mean ± SD *P < 0.05, **P < 0.01. 2-way ANOVA with Sidak’s multiple comparison (**C**–**F**) and unpaired two-tailed Student’s t-test (**H**–**L**) was used.

We previously reported that a *Col2-Cre* transgene targets articular and growth plate chondrocytes, synovial, meniscus and cruciate ligament cells in mice^11^. To generate mice with *Vegf*-deficient *Col2-Cre* expressing cells, we crossed female *Col2-Cre;Vegf* ^*fl*/+^ mice with male *Vegf* ^*fl/fl*^ or *Vegf* ^*fl*/+^ mice to generate *Col2-Cre;Vegf* ^*fl*/*fl*^ (*CKO* ^*Col2*^) mice (Table 1), because pups were hardly born when males carrying the *Col2-Cre* allele were used (Supplementary Table 1). Previous studies deleting *Vegf* ^*fl/fl*^ alleles in cells expressing collagen type II resulted in embryonic or postnatal lethality when males were used as carriers of the *Col2-Cre* transgene^9,12^. However, using a different *Col2-Cre* strain and selecting females as carriers of *Col2-Cre* for mating, viable, but not fertile, C*KO* ^*Col2*^ mice were generated. X-ray images showed that they were smaller than *Col2-Cre* controls at 4 weeks and 16 weeks (Fig. 1A and B). Body weights were reduced from 4 weeks to 10 weeks (Fig. 1C and D), and femurs and tibiae were shorter (Fig. 1E and F). Micro-CT showed low bone mass at 4 weeks (Fig. 1G). Tibial trabecular bone volume/tissue volume (BV/TV) and trabecular number (Tb.N) were markedly reduced (Fig. 1H and I). Trabecular spacing (Tb.Sp) was increased (Fig. 1J); however, trabecular thickness (Tb.Th) (Fig. 1K) and cortical thickness were not changed (Fig. 1L). Thus, VEGF in *Col2-Cre* lineage cells is important for accrual of trabecular bone mass; consistent with previous findings that over-expression of VEGF164, a major VEGF isoform, in *Col2-Cre* lineage cells results in increased bone mass^13^.

### VEGF is required for secondary ossification center (SOC) formation

Ossification of femoral and tibial epiphyses in *CKO* ^*Col2*^ mice was largely missing at 4 weeks, while formation of SOC in control mice was completed (Fig. 1M). The tibial epiphysis in *CKO* ^*Col2*^ mice was not replaced by bone tissue at 4 and 8 weeks of age (Fig. 1N), and it was completely lacking tartrate-resistant acid phosphatase (TRAP)-positive osteoclasts at 4 weeks (Fig. 1O). At 16 weeks, epiphyses in *CKO* ^*Col2*^ mice were replaced by bone, but articular surfaces and menisci were disorganized. Immunofluorescent staining of tibial sections showed that the epiphysis in C*KO* ^*Col2*^ mice was completely lacking CD31 positive cells at 4 weeks (Fig. 1P). Expression of the hypertrophic chondrocyte marker collagen X was stimulated in *CKO* ^*Col2*^ epiphyseal cells (Fig. 1Q), while VEGF expression was markedly reduced (Fig. 1R). These data indicate that VEGF stimulates cartilage vascularization and osteoclast formation during the formation of SOC; similar to what it is doing during endochondral bone development^12,14^.

### VEGF is a survival factor for growth plate chondrocytes

**Figure 2:**
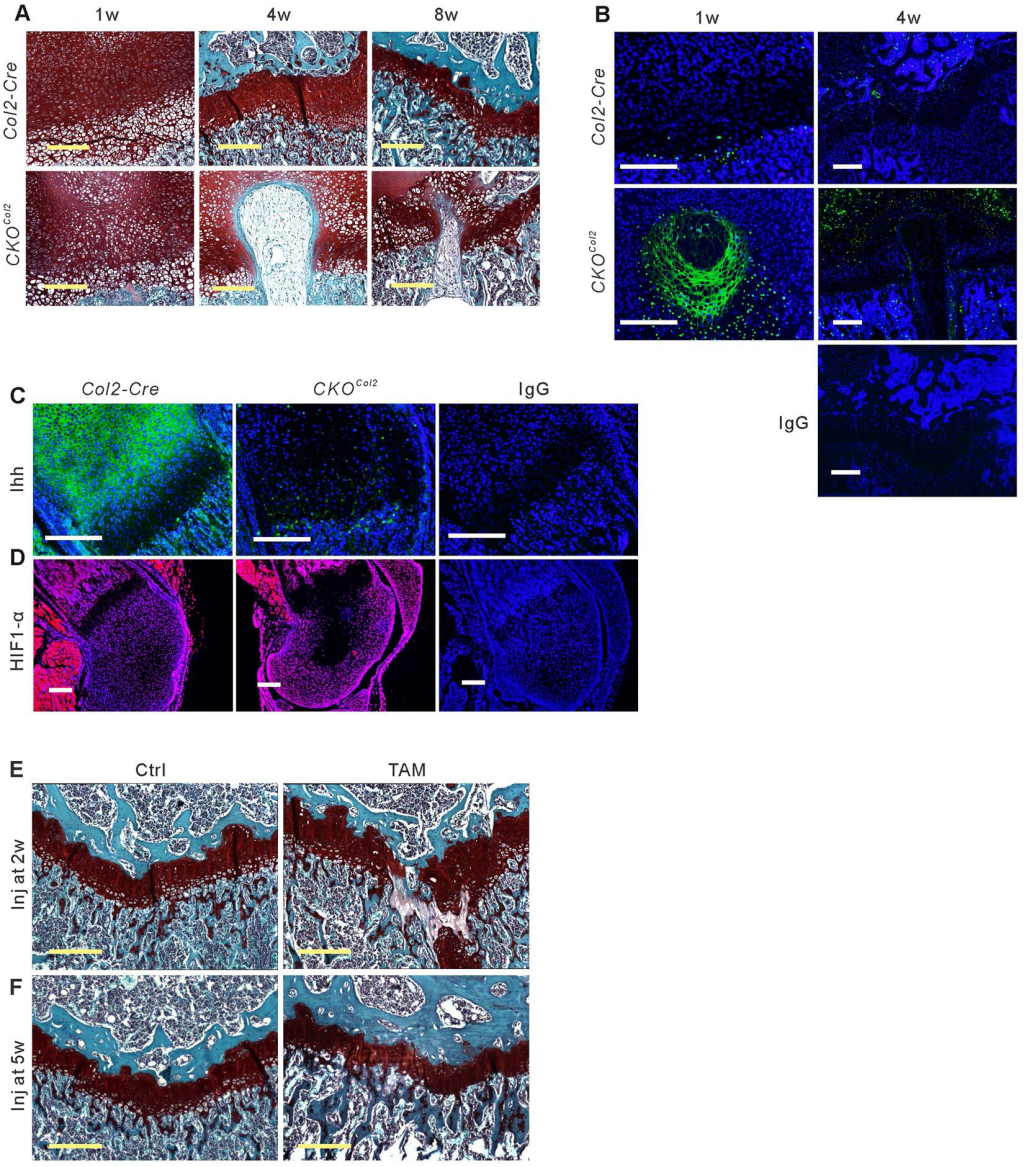
Attenuation of *Col2-Cre* lineage cell-derived VEGF induces cell death in growth plates until 4 weeks of age. Sections from femur of *Col2-Cre* and *CKO* ^*Col2*^ mice stained with Safranin O-fast green at 1, 4 and 8 weeks (w) of age (**A**) and TUNEL staining (green) at 1 and 4 weeks of age (**B**). Reduced immunofluorescence staining (green) for Indian Hedgehog (Ihh) in the tibial growth plate of mutants at 1 week of age (**C**), and reduced immunofluorescence staining (red) for HIF1-α in femoral epiphysis of mutants at 1 week of age (**D**); scale bars, 250um; DAPI (blue) used for nuclear staining. Sections of *Col2-Cre* mice used for control antibody (IgG) staining. Safranin O-fast green-stained sections of tibial growth plate from 9 weeks old mice show cell death in growth plates of *Col2-CreER;Vegf* ^*fl/fl*^ mice administered tamoxifen (TAM) intraperitoneally daily for 5 consecutive days at age 2 weeks (**E**) or 5 weeks (**F**), compared with mice administered corn oil (Ctrl). Scale bars, 250 μm.

In addition to structural abnormality in tibial epiphyses in *CKO* ^*Col2*^ mice, misshapen growth plates were observed (Fig. 2A); consistent with findings of apoptotic cell death^9^. Large numbers of TUNEL-positive cells were present at 1 week postpartum in *CKO* ^*Col2*^ mice, but only a few positive cells in the misshapen growth plate were observed at 4 weeks (Fig. 2B).

Indian hedgehog (Ihh), normally expressed in prehypertrophic chondrocytes and essential for growth plate maintenance^15^, were not seen in growth plate and epiphysis of *CKO* ^*Col2*^ mice (Fig. 2C). Expression of hypoxia-inducible factor 1α (HIF-1α) was also examined, since mice with HIF-1α-deficient chondrocytes exhibit massive apoptotic cell death in centrally located hypoxic regions in growth plates, similar to what is seen in mice with VEGF-deficient chondrocytes during development^9,16^. The number of cells expressing HIF-1α in growth plates and epiphyses was reduced in *CKO* ^*Col2*^ mice (Fig. 2D); however, considering that many cells in the center growth plate region underwent apoptosis, the level of expression in remaining live cells was comparable to that of control mice. Chondrocyte apoptosis in *CKO* ^*Col2*^ mice is therefore not an effect of reduced HIF-1α levels. To examine whether postnatal targeting of VEGF leads to chondrocyte apoptosis in growth plates, we injected tamoxifen in corn oil into *Col2-CreER;Vegf* ^*fl*/*fl*^ mice to generate *CKO* ^*Col2ER*^ mutant mice at 2 weeks of age and analyzed the mice 7 weeks later. Control *Col2-CreER;Vegf* ^*fl*/*fl*^ mice were injected with corn oil only (Table 1). In previous studies, we demonstrated that the *Col2-CreER* transgene in mice used for these experiments targets articular and growth plate cartilage, but not cells in synovium, meniscus and cruciate ligaments, when tamoxifen is given at 2 weeks of age^11^. Using *Col2-CreER;tdTomato* mice, these previous studies also demonstrated that when tamoxifen is administered at 2 weeks, Tomato fluorescence is detected in more than 80% of articular chondrocytes even 4 months after tamoxifen injection^11^. Body weights of *CKO* ^*Col2ER*^ mice were comparable to controls (Supplementary Fig. 1A and B). Tibia in mice given tamoxifen was 3–4% shorter, while femoral lengths were similar (Supplementary Fig. 1C and D). Histology showed misshapen growth plates (Fig. 2E). However, no misshapen growth plates were observed in *CKO* ^*Col2ER*^ mice at 9 weeks of age when tamoxifen was administered at 5 weeks (Fig. 2F). Therefore, VEGF, produced by *Col2-Cre* lineage cells, is not required as a survival factor in postnatal growth plate chondrocytes after 5 weeks of age.

To examine whether VEGF directly affects chondrocyte survival also *in vitro*, primary chondrocytes, isolated from postnatal *CKO* ^*Col2ER*^ and *Vegf* ^*fl/fl*^ mice, were exposed to 20% oxygen or 1% oxygen. LDH release, a measure of cytotoxicity, was increased in cultures of 1% oxygen compared with 20% oxygen (Supplementary Fig. 2). The numbers of TUNEL-positive adherent cells were extremely low (1% or less in both 1% and 20% oxygen cultures). Thus, mechanisms, underlying the survival effect of VEGF on growth plate chondrocytes during development and the early postnatal period, are unlikely to be cell autonomous, but involve cellular interactions on the tissue level.

### VEGF is required for articular chondrocyte differentiation

**Figure 3:**
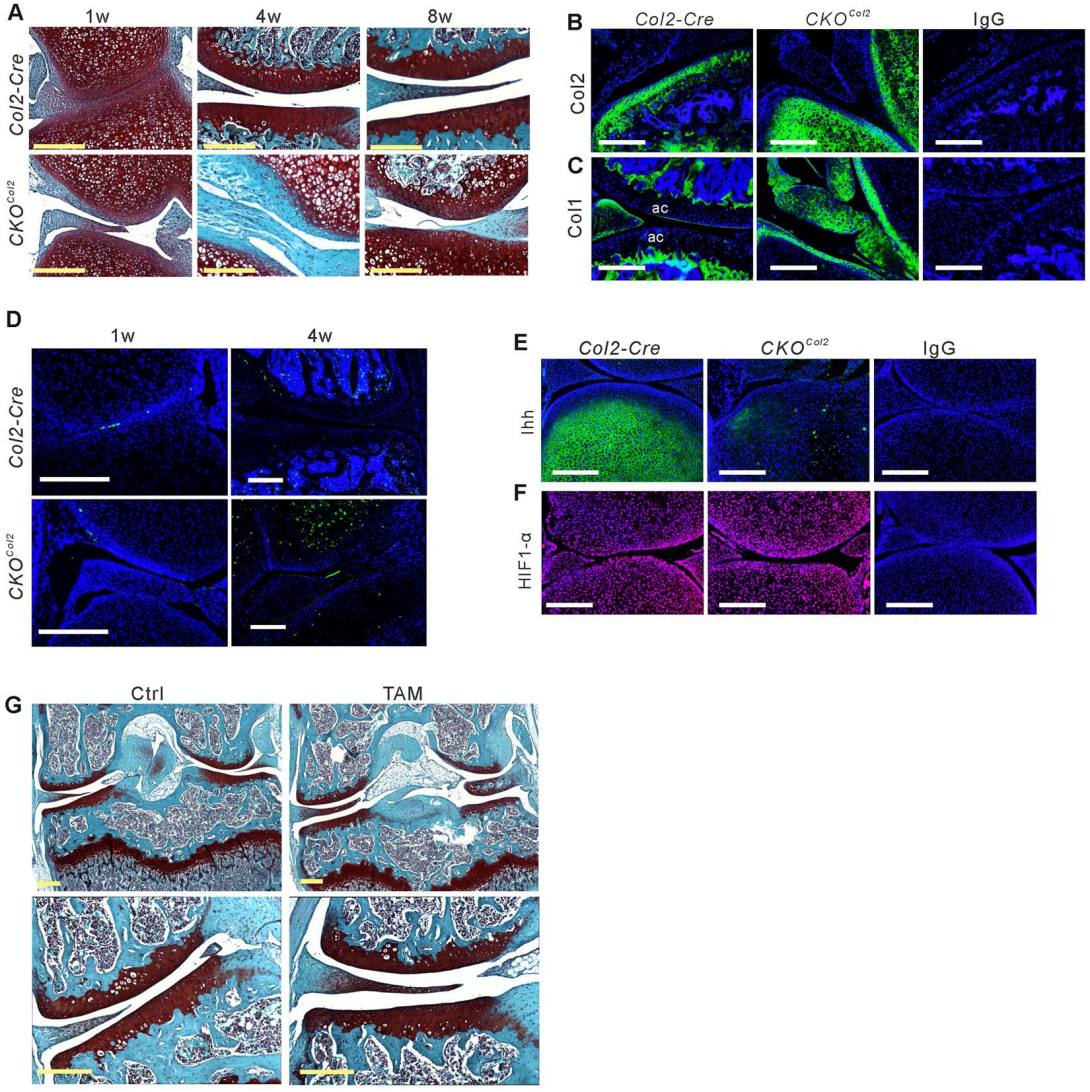
*Col2-Cre* lineage cell-derived VEGF is required for articular chondrocyte differentiation. Sections of knee joints in *Col2-Cre* and *CKO* ^*Col2*^ mice at 1, 4 and 8 weeks of age stained with Safranin O-fast green (**A**). Immunofluorescent staining (green) of sections of knee joints at 4 weeks for collagen II (Col2) (**B**) and collagen I (Col1) (**C**). Articular cartilage (ac) is not stained for collagen I in *Col2-Cre* control joint, but surface cells stain for collagen I in mutant joint (**C**). TUNEL staining (green) of sections of knee joints at 1 week and 4 weeks of age (**D**). Immunofluorescent staining of knee joint sections at 1 week for Indian hedgehog (Ihh, green) (**E**) and HIF-1α (red) (**F**). DAPI used for nuclear staining (blue). Safranin O-fast green-stained sections of knee joints from 9 weeks old *CKO* ^*Col2ER*^ mice administered corn oil (Ctrl) or tamoxifen (TAM) at 2 weeks of age daily for 5 consecutive days (**G**). Low magnification images are at top; higher magnification images are shown below. Scale bars, 250 μm.

Articular surface cells, showing greatly reduced Safranin-O-staining, were observed in *CKO* ^*Col2*^ mice at 1 and 4 weeks of age (Fig. 3A). At 8 weeks, the cells were Safranin-O-positive, but were morphologically different from articular surface cells in control mice (Fig. 3A). To characterize these cells, we stained sections with antibodies against collagen II, collagen I, Ihh and HIF-1α and for TUNEL-positivity. Collagen II was not expressed in *CKO* ^*Col2*^ surface cells at 4 weeks (Fig. 3B), but was expressed in articular cartilage of control mice. In contrast, collagen I was expressed in *CKO* ^*Col2*^ surface cells but not in controls (Fig. 3C). Articular cartilage in *CKO* ^*Col2*^ and *Col2-Cre* control mice contained only a few TUNEL-positive cells at 1 week, while many positive cells were observed in the epiphysis of *Col2-Cre* control mice and in the femoral epiphysis in *CKO* ^*Col2*^ mice at 4 weeks (Fig. 3D). Abundant Ihh expression was seen in control subchondral bone but not in *CKO* ^*Col2*^ and *Col2-Cre* mice (Fig. 3E). HIF-1α expression in the joint was similar in both phenotypes (Fig. 3F). These data suggest that *Col2-Cre* lineage cell-derived VEGF is not needed as a survival factor for articular chondrocytes, but is required for their differentiation. To examine whether VEGF has a role in maintaining the articular cartilage phenotype postnatally, we administered tamoxifen to *Col2-CreER;Vegf* ^*fl/fl*^ mice at 2 weeks and analyzed the mice 7 weeks later. In a previous study, we have shown that in these conditional *CKO* ^*Col2ER*^ mice, articular cartilage is targeted with high specificity^11^. When compared with control *Col2-CreER;Vegf* ^*fl*/*fl*^ mice, injected with corn oil, histology showed no changes in articular cartilage following treatment with tamoxifen (Fig. 3G), suggesting that postnatal deletion of VEGF has minimal effects on articular chondrocyte maintenance under physiological resting conditions.

### No effect of endothelial VEGF on growth plate and articular cartilage

To determine whether endothelial-derived VEGF affects growth plate chondrocyte survival, we generated *Flk1-Cre;Vegf* ^*fl/fl*^ (*CKO* ^*Flk1*^) mice (Table 1). The *Flk1-Cre* transgene specifically targets vascular endothelial cells^17^, but to confirm that this is indeed the case, we examined *Flk1-Cre;TdTomato* mice*. Tomato* expression in proximal tibia overlapped with antibody-staining for the endothelial marker endomucin (Supplementary Fig. 3A) and targeted cells were not overlapping with targeted cells in *Col2-Cre* mice (Supplementary Fig. 3B).

*CKO* ^*Flk1*^ mice were viable and fertile with body weights and leg lengths similar to controls (Supplementary Fig. 4A–D). Misshapen growth plates were not observed (Supplementary Fig. 5A) at 4 weeks, suggesting that VEGF produced by vascular endothelium does not contribute to VEGF function as a chondrocyte survival factor. Similarly, normal articular cartilage was observed in *CKO* ^*Flk1*^ mice at 4 and 8 weeks (Supplementary Fig. 5B). Thus, endothelium-derived VEGF does not contribute to differentiation of articular chondrocytes.

### VEGF levels and angiogenesis are increased in OA knee joints

**Figure 4:**
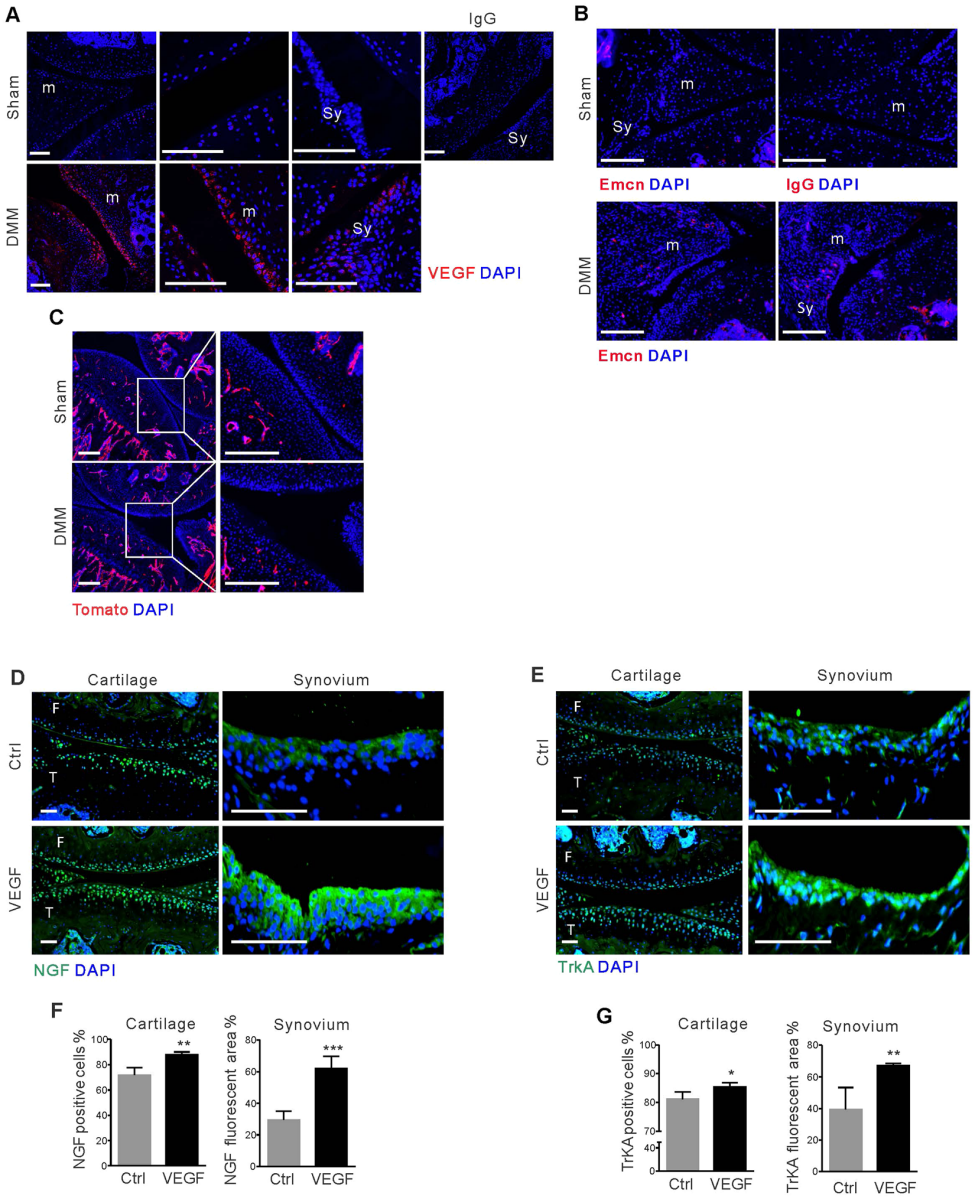
VEGF-dependent gene expression in chondrocytes and synovial cells and effects of DMM surgery. DMM surgery or sham surgery was performed in wild type (**A** and **B**) and *Flk1-Cre;TdTomato* mice (**C**) at 8 weeks of age. Sections of knee joints at 8 weeks (**A** and **B**) or 12 weeks (**C**) after DMM surgery with immunofluorescence staining (red) for VEGF (**A**), endomucin (**B**) or tdTomato (**C**); IgG, antibody control; m, meniscus; Sy, synovium; Emcn, endomucin. DAPI (blue) used for nuclear staining. Scale bars, 250 μm. PBS, with or without VEGF was injected into the knee joints of 24 week-old mice (n = 4) and tissues were harvested 3 days later. Immunofluorescence shows increased expression of NGF and TrkA in both articular cartilage and synovium (**D** and **E**) of joints injected with VEGF compared with PBS controls. DAPI (blue) used for nuclear staining. The percentages of NGF- and TrkA-positive cells were significantly increased in tissues exposed to VEGF (**F** and **G**). Data represent mean ± SD; *p < 0.05; **p < 0.01, ***p < 0.001; F: Femur; T: Tibia. Scale bars. 10 μm.

VEGF expression is increased in human OA knee joint tissues^18–20^. To further explore the role of VEGF in OA, we used a well-established mouse model (destabilization of the medial meniscus - DMM) of post-traumatic human knee OA. When DMM surgery was performed at 8 weeks of age and knee joints were examined eight weeks later, immunostaining showed increased levels of VEGF in articular cartilage, subchondral bone, meniscus and synovium (Fig. 4A). The VEGF staining-pattern, partially overlapping with nuclei of surface cells, was similar to staining patterns seen in bone marrow-derived Osterix-expressing cells, in which intracellular VEGF regulates their osteoblastic/adipogenic fates^21^. We also observed vascularization in meniscus and synovium of DMM mice, based on tomato expression in endothelial *Flk1-Cre* -positive lineage cells and endomucin antibody staining (Fig. 4B and C).

When VEGF was injected into the knee joints of control mice and joint tissues were collected 3 days later, both articular cartilage and synovial cells showed increased expression of nerve growth factor (NGF) and the receptor tropomyosin receptor kinase A (TrkA) (Fig. 4D–G). This is consistent with data indicating that synovial expression of NGF by fibroblasts and macrophages is increased in OA patients^21^.

### Reduction of *Vegf* expression attenuates OA progression

**Figure 5:**
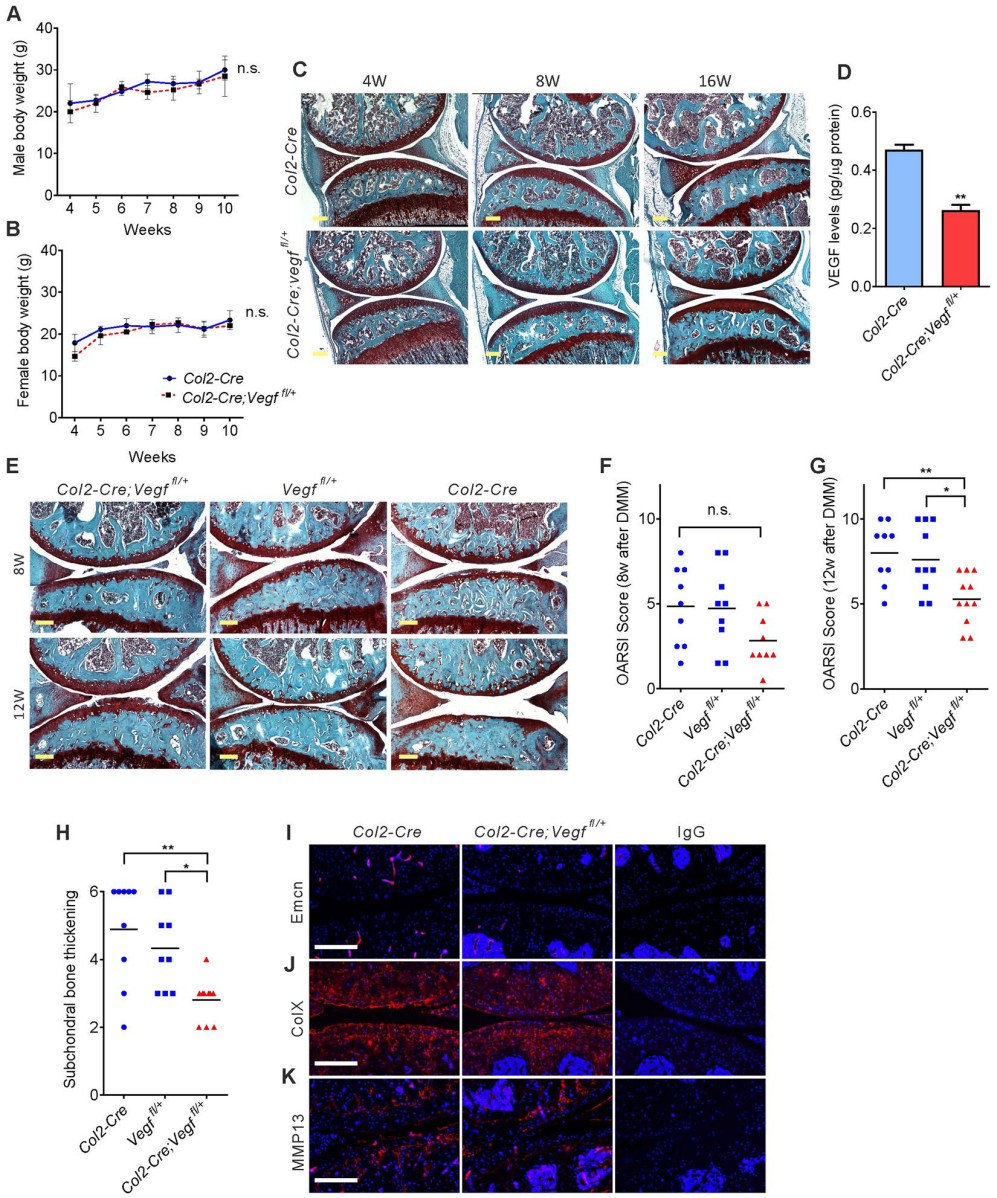
Reduction of *Vegf* in collagen type 2 (*Col2)* lineage cells attenuates OA progression. Body weights in male (**A**) and female (**B**) *Col2-Cre* or *Col2-Cre;vegf* ^*fl*/+^ mice from 4 to 10 weeks of age (*n* = 4–10). Safranin O-fast green staining of knee joint sections from 4, 8 and 16 week-old mice (**C**). ELISA assays showing reduced VEGF protein levels in lysates of mouse primary chondrocytes from heterozygous mutants (**D**). Safranin-O fast green-stained sections of knee joints 8 and 12 weeks after DMM surgery (**E**). Assessment of cartilage degradation by OARSI scores 8 weeks (*n* = 9) (**F**) and 12 weeks (*n* = 9–11) (**G**) after DMM surgery. Assessment of subchondral bone thickening 12 weeks after surgery (**H**) (*n* = 9–10). Immunostaining (red) of knee joint sections for endomucin (Emcn) (**I**), collagen X (ColX) (**J**) and MMP13 **(K)**, 12 weeks after DMM surgery. Scale bars, 250um; data represent mean ± SD; *P < 0.05 **P < 0.01. 2-way ANOVA with Sidak’s multiple comparison (**A** and **B**), unpaired Student’s t-test (**D**) and one-way ANOVA with Tukey’s multiple comparison (**F**–**H**) were used.

To determine whether reduction of VEGF expression may attenuate OA progression, knee joints of mice haplo-insufficient for *Vegf* in *Col2* lineage cells (*Col2-Cre;Vegf* ^*fl*/+^) and two control groups, *Vegf* ^*fl*/+^ and *Col2-Cre*, were compared (Table 1). In contrast to the severe defects in early development of *CKO* ^*Col2*^ mice, *Col2-Cre;Vegf* ^*fl*/+^ mice exhibited normal body weights (Fig. 5A and B) and had no morphological knee joint abnormalities (Fig. 5C) when compared to controls, although VEGF levels were reduced about 45% as determined by ELISA assays of lysates of primary chondrocytes isolated from *Col2-Cre* and *Col2-Cre;Vegf fl/*+ mice (Fig. 5D). Micro CT analysis indicated slightly reduced BV/TV in *Col2-Cre Vegf* ^*fl*/+^ male mice; however, other bone parameters were not changed (Supplementary Table 2). DMM surgery was performed in *Col2-Cre;Vegf* ^*fl*/+^, *Vegf* ^*fl*/+^ and *Col2-Cre* mice, and mice were analyzed 8 and 12 weeks later. OA progression, assessed by Osteoarthritis Research Society International (OARSI) grading, was attenuated 12 weeks after surgery in *Col2-Cre;Vegf* ^*fl*/+^ mice compared to *Vegf* ^*fl*/+^ and *Col2-Cre* control mice, while differences 8 weeks after surgery were not significant (Fig. 5E–G). Thickening of subchondral bone was also reduced in *Col2-Cre;Vegf* ^*fl*/+^ mice (Fig. 5H). Immunostaining for endomucin showed reduced neovascularization of subchondral bone in *Col2-Cre;Vegf* ^*fl*/+^ mice, while collagen X expression was similar in the mutant and the *Col2-Cre* control groups (Fig. 5I and J). Immunostaining for MMP13 showed reduced expression in articular cartilage and subchondral bone (Fig. 5K). These data suggest that VEGF expression in *Col2-Cre* lineage cells contributes to OA progression and that deletion of one *Vegf* allele in the cells is sufficient to attenuate this progression.

### VEGF in cartilage and endothelium does not affect OA progression

**Figure 6:**
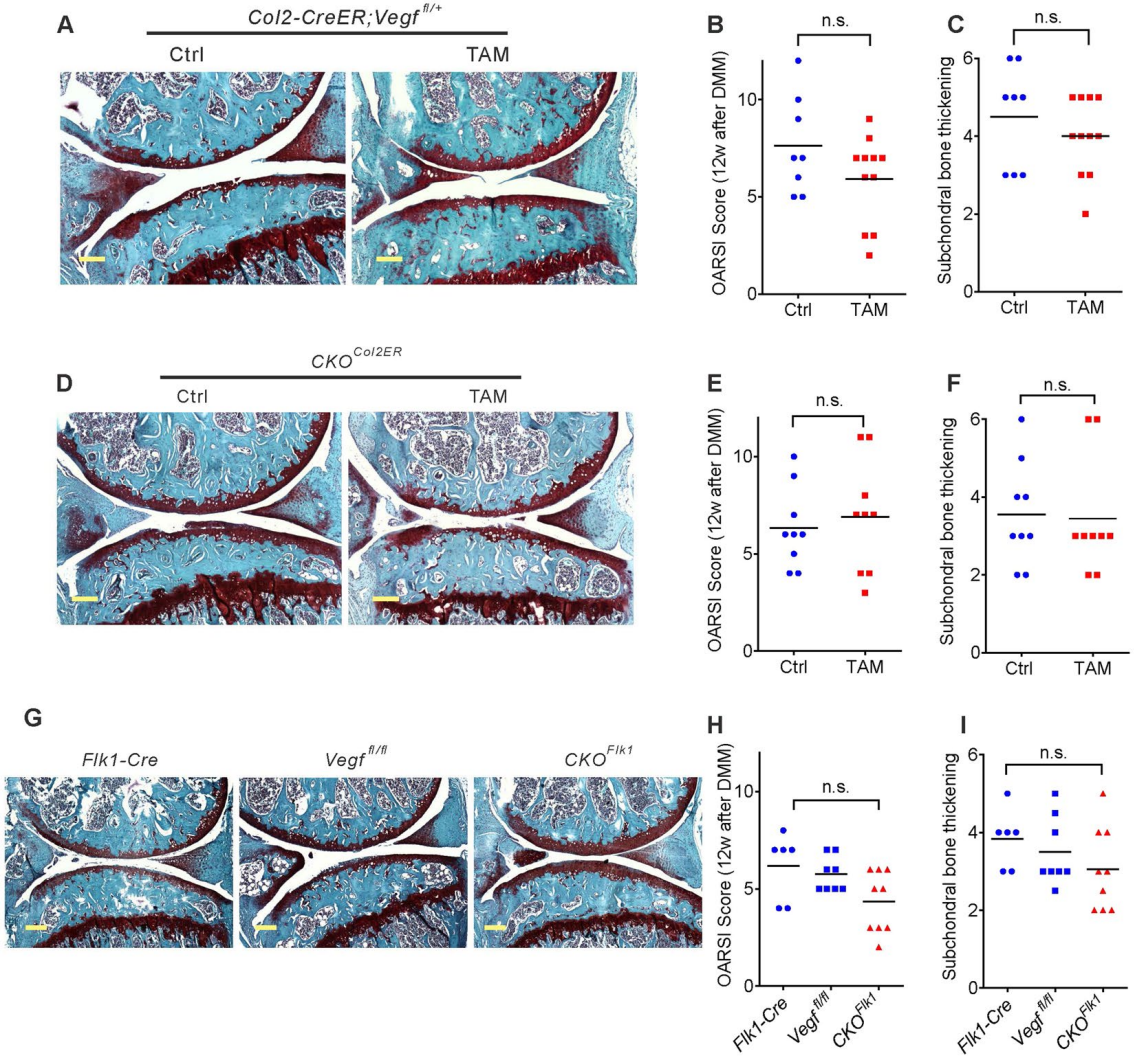
Deletion of both *Vegf* alleles in tamoxifen-induced *Col2-CreER* mice does not attenuate OA progression. Safranin O-fast green-stained sections of knee joints from *Col2-CreER;Vegf* ^*fl*/+^ mice administered corn oil (Ctrl) or tamoxifen (TAM) at 2 weeks of age, subjected to DMM surgery at 8 weeks and analyzed at 20 weeks (**A**). Assessment of cartilage degradation by OARSI grading (**B**) and subchondral bone thickening (**C**) (*n* = 8 and 11). Safranin O-fast green-stained sections of knee joints from *Col2-CreER;Vegf* ^*fl/fl*^ (*CKO* ^*Col2ER*^) mice treated in the same way, subjected to DMM surgery at 8 weeks and analyzed at 20 weeks (**D**). Assessment of cartilage degradation by OARSI grading (**E**) and subchondral bone thickness (**F**) (*n* = 9). Sections of knee joints, stained with Safranin O-fast green, from *Flk1-Cre;Vegf* ^*fl/fl*^ (*CKO* ^*Flk1*^) and *Flk1-Cre* and *Vegf* ^*fl/fl*^ (2 controls) mice after DMM surgery at 8 weeks of age and analyzed at 20 weeks (**G**). Assessment of cartilage degradation by OARSI grading (**H**) and subchondral bone thickness (**I**) (*n* = 6–9). Scale bars, 250 μm; data represent mean ± SD; *P < 0.05. Unpaired Student’s t-test (**B**,**C**,**E** and **F**) and one-way ANOVA with Tukey’s multiple comparison (**H** and **I**) were used.

When *Col2-Cre* is used for targeted deletion of one *Vegf* allele, reduction of VEGF expression occurs in all *Col2-Cre* expressing progenitors and their lineage cells, including cells in subchondral bone, meniscus and cruciate ligaments, as well as articular chondrocytes and synovial cells. In contrast, when induced with tamoxifen 2 weeks after birth, the *Col2-CreER* transgene primarily targets articular chondrocytes, is less effective in the meniscus and bone and does not target synovium and cruciate ligament^11^. To assess the contribution of articular chondrocyte-derived VEGF to OA progression, we administered tamoxifen in corn oil to 2-week old *Col2-CreER;Vegf* ^*fl*/+^ and *Col2-CreER;Vegf* ^*fl/fl*^ (*CKO* ^*Col2ER*^) mice, carrying one or two floxed *Vegf* alleles, respectively (Table 1). Controls were injected with corn oil only. DMM surgery was performed at 8 weeks and the knee joint analyzed at 20 weeks. Mean body weights in the two groups were similar to the controls before and after surgery (Supplementary Fig. 6A–D). Cartilage degradation, assessed by Safranin O-fast green staining, OARSI grading and measurements of subchondral bone thickness, were not significantly different from the controls when one (Fig. 6A–C) or two (Fig. 6D–F) *Vegf* alleles were targeted. Therefore, depletion of *Vegf* synthesis in articular chondrocytes only is not sufficient to affect OA progression.

To determine whether lack of VEGF-synthesis in endothelial cells may contribute to attenuation of OA progression, *CKO* ^*Flk1*^ mice were analyzed 12 weeks after DMM surgery at 8 weeks. The female *CKO* ^*Flk1*^ mice had similar body weights as controls, while male *CKO* ^*Flk1*^ mice had reduced body weights (Supplementary Fig. 6E and F). OA progression in *CKO* ^*Flk1*^ mice was slightly attenuated compared to control *Vegf* ^*fl*/+^ or *Flk1-Cre* mice; however, the differences were not significant by univariate analysis (Fig. 6G–I). After adjustment for body weight by multiple regression analysis, the association between *Flk1-Cre* and *CKO* ^*Flk1*^ (R-squad = 0.29, P value = 0.08) or *Vegf* ^*fl*/+^ and *CKO* ^*Flk1*^ (R-squad = 0.25, *P* value = 0.12) was not significant. These results suggest that reducing VEGF synthesis in the endothelium does not attenuate OA progression to a significant degree.

### Intra-articular VEGF antibody treatment reduces OA progression

**Figure 7:**
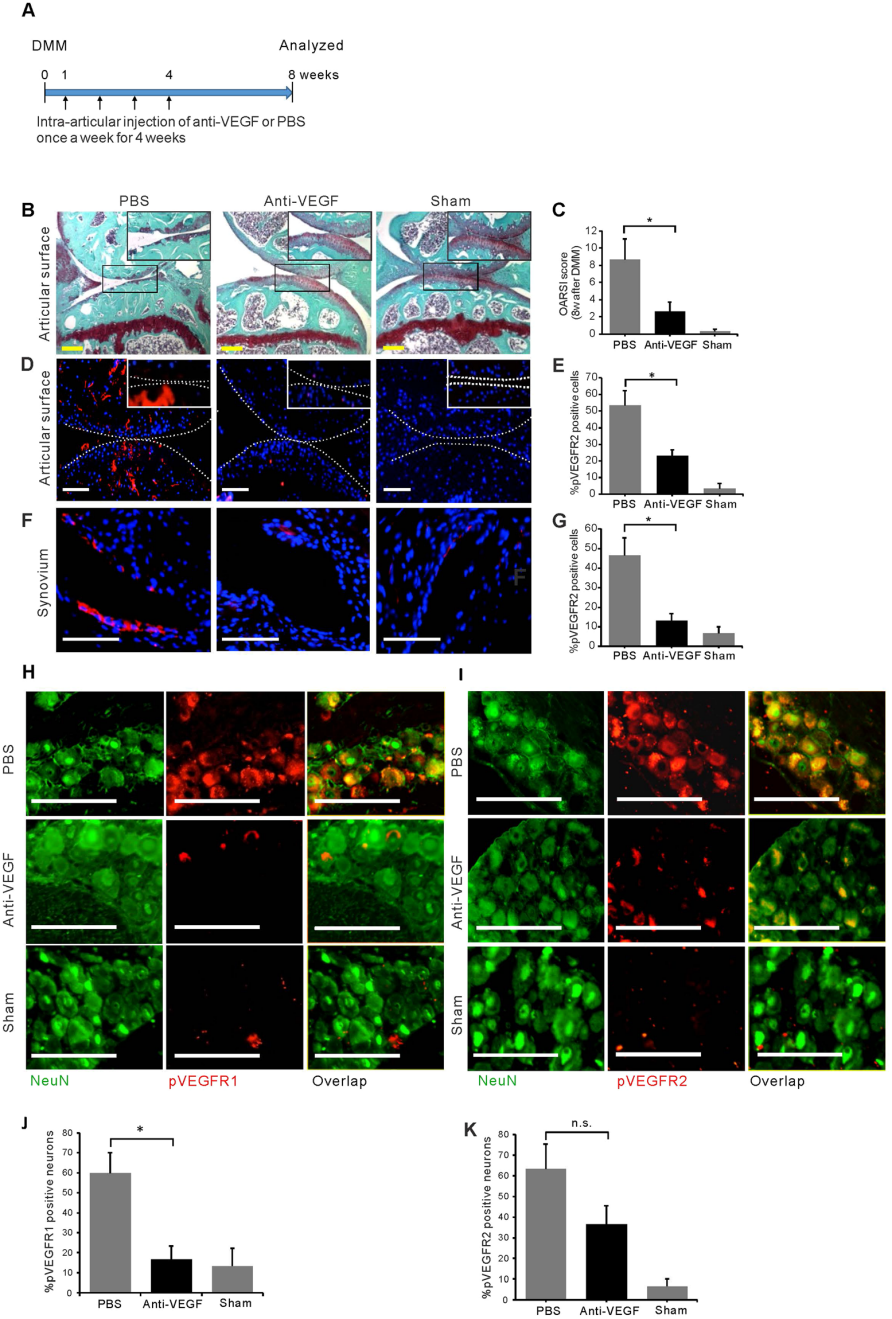
Effects of VEGF on OA progression. DMM or sham surgery was performed in 24 week-old wild type C57BL/6J mice and the outcome analyzed 8 weeks later. The mice received intra-articular anti-VEGF antibody or phosphate buffered saline (PBS) at weeks 1, 2, 3 and 4 post DMM (**A**) (*n* = 10). Sections of knee joints were stained with Safranin O-fast green, high magnification insets show articular cartilage details (**B**). Assessment of cartilage degradation by OARSI grading scores (*n* = 6) (**C**). Immunofluorescence staining of phosphorylated VEGFR2 (pVEGFR2) close to the articular surface; high magnification inset (**D**), in synovium (**F**) and dorsal root ganglia (DRG) (**I**). Immunofluorescence staining for phosphorylated VEGFR1 (pVEGFR1) in the DRG (**H**); numbers of pVEGFR2 positive cells in articular surface region (**E**) and synovium (**G**) are reduced in anti-VEGF treated joints. Numbers of pVEGFR1 positive cells in DRG neurons are significantly reduced in anti-VEGF treated joints (**J**); reduction of pVEGFR2 positive cells is not significant (**K**). Red fluorescence represents pVEGFR2 (**D,F** and **I**) or pVEGFR1 (**H**); green fluorescence represents neuronal nuclear antigen (NeuN) (**H** and **I**); blue represents DAPI nuclear staining. Scale bars, 250 μm; *P < 0.05. Data represent mean ± SEM. Unpaired Student’s t-test was used.

Our genetic studies demonstrate that targeting VEGF in *Col2-Cre* lineage cells, such as cells in meniscus, synovium, ligaments and subchondral bone, may be beneficial in attenuating OA progression. However, the genetic approach targets synthesis of both intra- and extracellular VEGF, and which of the two forms of VEGF needs to be targeted is not clear. Therefore, to examine whether targeting extracellular VEGF in the knee joint could be beneficial, 24 week-old wild type mice received intra-articular anti-VEGF antibody or phosphate buffered saline (PBS). Intra-articular injections were given once a week, starting one week after DMM or sham surgery and knee joints were analyzed 8 weeks later (Fig. 7A). Cartilage degradation was reduced in the anti-VEGF group (Fig. 7B), and quantification of OA severity showed that mice receiving anti-VEGF antibody exhibited attenuated OA progression compared to the PBS group (Fig. 7C).

To examine downstream effects of anti-VEGF treatment, we analyzed phosphorylated VEGF receptor 2 (pVEFR2) expression levels. Immunofluorescence of the articular surface and synovium showed that levels of pVEGFR2 in the anti-VEGF group were dramatically reduced (Fig. 7D and F) and the percentage of articular chondrocytes and synovial cells co-stained with DAPI and anti-pVEGFR2 were reduced (Fig. 7E and G). These data suggest that inhibiting extracellular VEGF function attenuates OA progression and reduces VEGFR2 signaling in articular chondrocytes and synovial cells of mice with DMM-induced OA.

Interestingly, when we analyzed pVEGFR1 and pVEGFR2 expression in L3–5 dorsal root ganglion cells (DRG) ipsilateral to the site of DMM, the percentage of anti-pVEGFR1 positive cells was significantly reduced in the anti-VEGF group (Fig. 7H and J). Anti-VEGF treatment appeared to also reduce pVEGFR2 expression in DRG; however, this reduction was not statistically significant (Fig. 7I and K). These data suggest that inhibiting extracellular VEGF function in the OA knee joint may have effects on DRG as well.

### VEGFR2 kinase inhibitor attenuates OA progression

**Figure 8:**
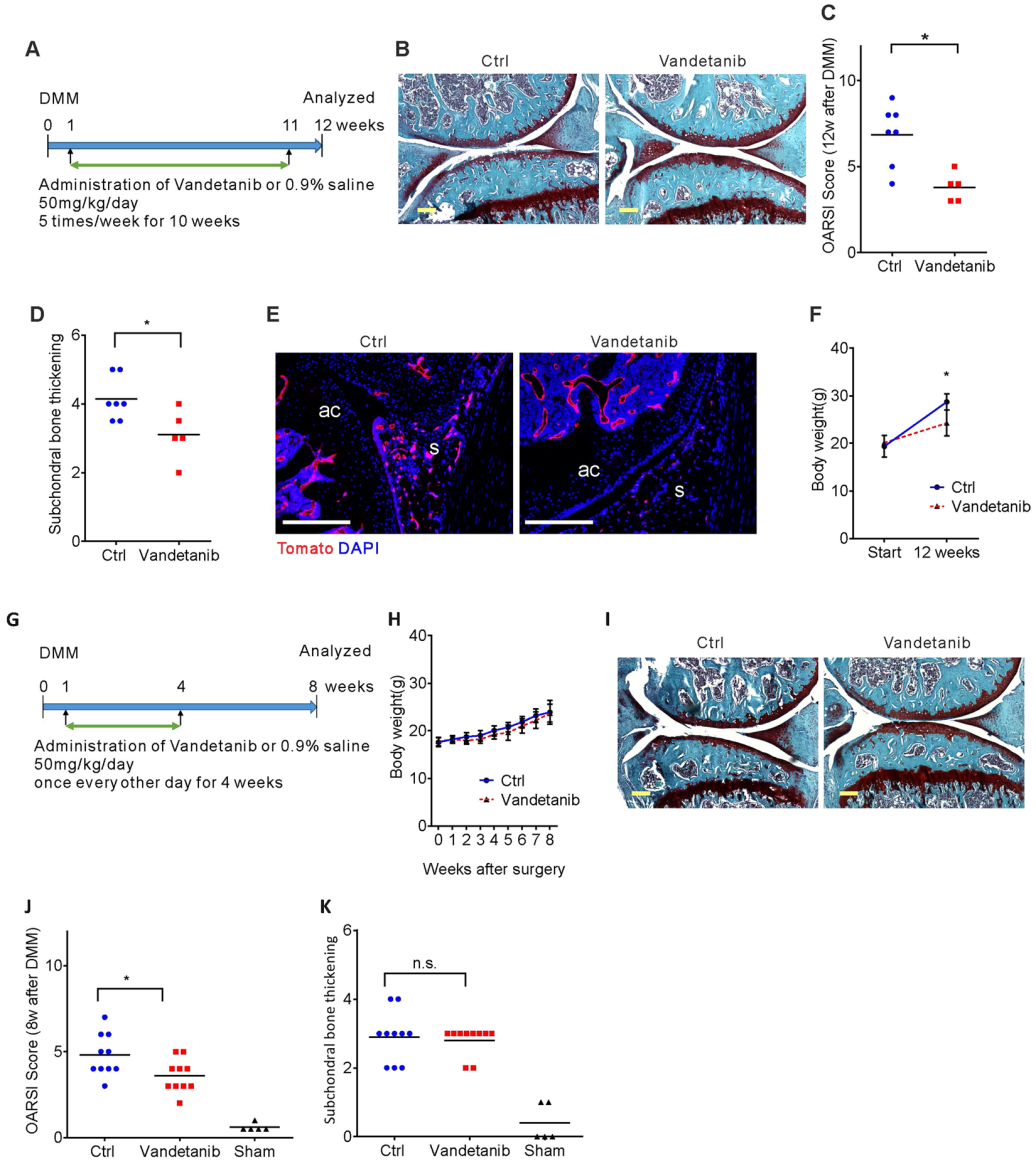
Oral administration of VEGFR2 kinase inhibitor attenuates OA progression. DMM or sham surgery was performed in 8 week-old *Flk1-Cre;tdTomato* (**A–E**) mice and the outcome analyzed 12 weeks after surgery (*n* = 5–7). The mice received VEGFR2 tyrosine kinase inhibitor, Vandetanib, or phosphate buffered saline (PBS) for 10 weeks (**A**). Sections of knee joints were stained with Safranin O-fast green (**B**). Assessment of cartilage degradation by OARSI grading (**C**) and subchondral bone thickness (**D**) showed attenuation of OA in Vandetanib-treated animals. Immunofluorescence images for Tomato (red) in the synovium (s); articular cartilage (ac); DAPI staining blue (**E**). Body weights before and 12 weeks after surgery (*n* = 5–7) (**F**). DMM or sham surgery was performed in 8 week-old wild type (**G–K**) mice and outcomes were analyzed 8 weeks after surgery. The mice received Vandetanib or PBS for 4 weeks (**G**). Body weights before and 8 weeks after surgery (*n* = 10) (**H)**. Sections of knee joints stained with Safranin O-fast green (**I**). Assessment of cartilage degradation by OARSI grading (**J**) showed attenuation of OA in Vandetanib-treated animals, but subchondral bone thickness was not affected (**K**). Scale bars, 250 μm; *P < 0.05. Data represent mean ± SD. Unpaired Student’s t-test (**C,D,J** and **K**) and 2-way ANOVA with Sidak’s multiple comparison (**F** and **H**) were used.

Reduction of pVEGFR2 levels in synovium and articular cartilage in DMM mice treated with anti-VEGF antibodies suggests that inhibition of VEGFR2 signaling may also affect progression of OA. Oral administration of Vandetanib (ZD6474), an FDA approved small molecule tyrosine kinase inhibitor of VEGFR2^22^, has been demonstrated to inhibit tumor angiogenesis by decreasing endothelial cell proliferation and VEGF-dependent endothelial cell survival^23^. Vandetanib administration also produces dose-dependent hypertrophy of growth plates in young rodents^23^. To determine whether Vandetanib may attenuate OA progression in mice with DMM-induced OA, we first performed a pilot study. 50 mg kg^−1^ Vandetanib or 0.9% saline was given 5 times a week for 10 weeks, starting one week after DMM surgery, to female *Flk1-Cre;TdTomato* mice, similar to what has been done in previous studies^23^. The mice were analyzed 12 weeks after surgery (Fig. 8A). No mice died during the pilot study, and OA progression, assessed by OARSI grading as well as subchondral bone thickening, was attenuated in Vandetanib-treated mice (Fig. 8B,C and D). Endothelial cell numbers in the control OA synovium were also reduced in the Vandetanib group (Fig. 8E). Since body weights were significantly reduced in Vandetanib treated mice (Fig. 8F), we changed the dosage and administered 50 mg kg^−1^ Vandetanib once every other day for 4 weeks (Fig. 8G). At 8 weeks after DMM surgery, the body weights of Vandetanib and control OA mice were similar (Fig. 8H), and no mice died during the experiment. Similar to the pilot study, OA progression, assessed by Safranin O-fast green staining and OARSI grading, in Vandetanib treated mice was attenuated, while subchondral bone thickening did not change (Fig. 8I,J and K).

## Discussion

VEGF has distinct roles in cartilage development and postnatal homeostasis. In growth plates, VEGF functions as survival factor for chondrocytes in hypoxic regions during development and early postnatal life, and its expression by hypertrophic chondrocytes is crucial for timely ossification of both primary and secondary centers in endochondral bones. In articular chondrocytes it is needed for differentiation to *Col2a1*-expressing cells, but it is hardly expressed in articular cartilage of young mice and appears not to play a significant role in maintaining articular cartilage after it is formed. A large fraction, 90%, of articular chondrocytes are targeted in *CKO* ^*Col2ER*^ mice when tamoxifen is injected at 2 weeks of age, and the remaining 10% VEGF is sufficient to maintain the cartilage. Several molecules, including Wnt family members and GDF5 are essential for articular cartilage formation^24–26^, but VEGF is somewhat unique in that it appears to have a different role in growth plate and articular chondrocytes.

HIF-1α and VEGF coordinately act to maintain cartilage metabolism under hypoxic conditions^10,27^. We observed reduced HIF-1α expression in *Vegf-*deficient growth plates and epiphyses; however, the pattern of expression in live cells around areas of massive cell apoptosis was similar to that of control mice one week after birth. The data are consistent with a role for *HIF-1α* as a regulator of oxygen consumption in chondrocytes by VEGF-independent mechanisms^13^. A similar *HIF-1α* expression pattern is seen in *Vegf-*deficient and control articular cartilage, suggesting that HIF-1α is not involved in mechanisms underlying VEGF-dependent articular chondrocyte differentiation.

*Vegf*-deficient articular surface cells in *CKO* ^*Col2*^ mice express collagen I, but not collagens II and X, during the first month after birth. Whether the effect of VEGF on articular chondrocyte differentiation is mediated by its interaction with VEGF receptors is not known. A previous study showed that VEGFR2 expression is undetectable in developing cartilage^10^, and mice with VEGFR2 conditionally knocked down in *Col2-Cre* lineage cells have no noticeable embryonic abnormalities^13^. The present study confirms that epiphyseal chondrocytes from newborn mice do not express VEGFR2. Although these findings suggest that effects of VEGF may not be mediated by VEGFR2, it is still possible that even low levels of the receptor or other receptors, such as VEGFR1, may contribute to development of articular cartilage. Notably, not all articular surface cells in *CKO* ^*Col2*^ mice appear to be undifferentiated; lateral condyle chondrocytes are mostly undifferentiated, the condyle is not well formed and appears to be reduced in its load bearing capacity compared with control mice. Since mechanical forces acting on articular cartilage stimulate VEGF expression^28,29^, VEGF may regulate articular chondrocyte differentiation in response to mechanical forces.

Chondrocyte hypertrophy is a key step in endochondral ossification and has been associated with OA. Hypertrophic chondrocytes secrete collagen X along with other factors that promote matrix mineralization and ossification^30^, and current understanding is that chondrocyte hypertrophy in OA articular cartilage is associated with mineralization. It is likely that VEGF does not regulate chondrocyte hypertrophy directly, but affects mineralization/ossification through an indirect mechanism. During development, the epiphysis in *Vegf*-deficient *CKO* ^*Col2*^ mice shows increased collagen X expression and less angiogenesis; consequently, formation of the SOC is delayed. Consistent with this is the finding that *Runx2*-deficient mice exhibit robust chondrocyte hypertrophy without VEGF expression and bone formation^31^.

In the present study, mice lacking VEGF-synthesis in vascular endothelial cells exhibited no detectable skeletal growth defects and showed no signs of early lethality, at least until 10 weeks of age. In contrast, mice lacking VEGF in *VE-Cadherin-Cre* lineage cells have a shortened life span^32^. The difference in the life span of the two vascular endothelial VEGF-deficient mice may be due to differences in *Cre*-targeted cells. The *Flk1-Cre* transgene used in the present study targets endothelium only^33^, whereas *VE-Cadherin-Cre* may target hematopoietic and lymphatic endothelial cells in addition to vascular endothelium.

In human OA patients, expression of VEGF has been found to be increased in articular cartilage, synovium and synovial fluid, subchondral bone and serum (for references, see^5^), and increased levels of VEGFR1 and VEGFR2 have been reported in OA chondrocytes^18^. Our data indicate that deleting VEGF in *Col2-Cre* lineage cells attenuates progression of surgically induced OA in mice, and reduces thickening of subchondral bone. The effect on subchondral bone is consistent with studies showing that VEGF stimulates subchondral bone thickening and induces OA in mice^6,34^. Previous studies also showed that VEGF stimulates osteoblastic differentiation^21,35^. In the present study, subchondral bone thickening was positively correlated with OARSI grade, but was independent of VEGF expression levels (Supplementary Table 3). Therefore, the effect of VEGF on subchondral bone may be an indirect consequence of its effects on articular cartilage.

Targeting VEGF in *Col2-Cre* lineage cells attenuated OA progression, but targeting the growth factor in *CKO* ^*Col2ER*^ mice, with VEGF reduced only in articular chondrocytes, and *Flk1-Cre* mice, with VEGF reduced only in vascular endothelial cells, did not. The finding that intra-articular anti-VEGFA antibody treatment attenuated OA progression indicates that extracellular VEGF generated by other cell types in the joint (i.e., fibroblast-like synovial cells, synovial macrophages, meniscal fibro-chondrocytes or cells in subchondral bone) is primarily responsible for promoting the OA process.

Intra-articular anti-VEGF antibody treatment reduced levels of activated pVEGFR2 in both articular chondrocytes and synovial cells. OA chondrocytes exhibit significant upregulation of VEGFR2, while non-OA chondrocytes have minimal VEGFR2 expression^18^. Our data, showing that systemic administration of Vandetanib reduces progression of OA, are consistent with the possibility that increased signaling via VEGFR2 plays a role in promoting cartilage degradation in OA. In addition to inhibiting the kinase activity of VEGFR2, Vandetanib also inhibits epidermal growth factor receptor (EGFR) and VEGFR1, but the selectivity for EGFR and VEGFR1 was reported to be 12.5 and 40 times less than for VEGFR2^22^. Systemic Vandetanib treatment was not as effective in reducing progression of OA as intra-articular administration of anti-VEGF antibody. One explanation may be that local delivery of a drug is more potent than systemic administration^36^. We were unable to examine the effects of intra-articular injection of Vandetanib because of its insolubility in PBS or 0.9% saline. Another explanation may be that anti-VEGF antibody treatment inhibits VEGF-mediated signaling via both VEGFR1 and VEGFR2 receptors, reducing NGF levels, and thus elicits a more beneficial effect.

Such secondary effects may also explain the remarkable efficiency of weekly intra-articular injections of the antibodies. Both VEGFR1 and VEGFR2 are present in the soma of DRG neurons^37^. Our data show that intra-articular treatment with anti-VEGF antibody significantly reduced pVEGFR1 levels and lowered levels of pVEGFR2 in the DRG. Since anti-VEGF antibody was administered early, starting 1-week post DMM, it was not possible to determine whether anti-VEGF antibody treatment resulted in reduced phosphorylation of the two VEGF receptors more directly or reduced activation of the receptors in DRG by inhibiting joint degeneration. However, our data indicate that VEGF stimulates chondrocytic and synovial expression of NGF. Therefore, intra-articular anti-VEGF antibody treatment not only attenuates OA joint degeneration, it may also lower NGF levels in joint tissues and reduce activation of VEGF receptors in DRG cells.

## Methods

### Animals

*Col2-Cre* ^38^, *Vegf receptor type 2 (Flk1)-Cre* ^17^ and *floxed-Vegf* ^39^ mouse lines have been described previously. *Col2-CreER* mice^40^ and *Rosa-TdTomato* mice were purchased from The Jackson Laboratories. *Cre* recombination efficiency was evaluated by *TdTomato* fluorescence in *Col2-Cre* mice or *Col2-creER* mice that were mated with *Rosa-TdTomato* reporter mice as published elsewhere^11^. To generate *Col2-Cre;Vegf* ^*fl/fl*^ (*CKO* ^*Col2*^) mice, female *Col2-Cre;Vegf* ^*fl*/+^ mice were crossed with male *Vegf* ^*fl*/+^ or *Vegf* ^*fl/fl*^ mice. To generate *Col2-CreER;Vegf* ^*fl/fl*^ (*CKO* ^*Col2ER*^) mice, male *CKO* ^*Col2ER*^ mice were crossed with female *Vegf floxed* mice. Genomic DNA isolated from portions of mouse tails were used for genotyping. The sequence of PCR primers for *Vegf* are: forward 5′-CCTGGCCCTCAAGTACACCTT-3′; reverse 5′-TCCGTACGACGCATTTCYAG-3′. The sequence of PCR primers for *Cre* are: forward 5′-GAACCTGATGGACATGTTCAGGGA-3′; reverse 5′-CAGAGTCATCCTTAGCGCCGTAAA-3′. The sequence of PCR primers for *Flk1-Cre* are: forward 5′-AAGGAGTCTGTGCCTGAGAAC-3′; reverse 5′ CTAGAGCCTGTTTTGCACGTTC-3′. The sequence of PCR primers for TdTomato are: forward 5′-CTGTTCCTGTACGGCATGG-3′; reverse 5′-GGCATTAAAGCAGCGTATCC-3′. In using animals for experiments, no blinding or randomization could be done because mice with different specific genotypes were compared. The number of animals in each experimental group was based on previous studies and pilot experiments.

All animal experiments were performed according to protocols approved by the Harvard Medical Area Standing Committee on Animals in accordance with U.S. Public Service Policy on Humane Care and Use of Laboratory Animals or by the Rush University Medical Center Institutional Care and Use Committee.

### *CreER* activation by tamoxifen

Tamoxifen (T5684, Sigma-Aldrich) was dissolved in corn oil at a concentration of 20 mg per ml by shaking overnight at 37 °C and injected intraperitoneally in 2 or 5 weeks old *Col2-CreER;Vegf* ^*fl*/+^ *and CKO* ^*Col2ER*^ mice. Animals received 75 mg tamoxifen per kg body weight once every 24 hours for a total of 5 consecutive days. Tamoxifen-oil mixture was stored at −20 °C until used.

### Surgical OA and anti-VEGF and VEGFR2 inhibitor treatment

To surgically induce OA in *Vegf* ^*fl*/+^, *Col2-Cre;Vegf* ^*fl*/+^, *Col2-Cre, Col2-CreER;Vegf* ^*fl*/+^, *CKO* ^*Col2ER*^, *CKO* ^*Flk1*^, *Flk1-Cre* mice and healthy control mice, 8 to 9 weeks old mice were used. Mice were anesthetized by intraperitoneal injection of Ketamine (100 mg per kg) and Xylazine (10 mg per kg) and knee regions were prepared for aseptic surgery. Experimental OA was induced by destabilization of the medial meniscus (DMM surgery)^41^. Briefly, after sedation, the joint capsule immediately medial to the patellar tendon was incised and the medial meniscotibial ligament (MMTL) of the medial meniscus was exposed. Sectioning of MMTL resulted in destabilization of the medial meniscus. Sham surgery was performed on the contralateral knee, except in the case of mice treated with intra-articular VEGF antibody. For anti-VEGF treatment, 24 week-old C57BL/6 J female mice were used. The mice received intra-articular injection of 5 μL of 1 mg/mL anti-VEGF antibody (2G11-2A05, BioLegend) or 5 μL of intra-articular phosphate buffered saline (PBS) at weeks 1, 2, 3 and 4 post DMM surgery; sham surgery was performed on different control mice. Mice were analyzed 8 weeks after DMM surgery. For VEGFR2 tyrosine kinase inhibitor, Vandetanib (ZD6474, S1046, Selleckchem) treatment, 8 weeks old female *Flk1-Cre;TdTomato* or C57BL/6 J wild type mice purchased from the Charles River Laboratories were used for DMM surgery in the right knee joint and sham surgery in the left joint. 50 mg per kg Vandetanib was administered, starting a week after surgery, once a day for 10 weeks (pilot study) or once every other day for 4 weeks. Vandetanib, an orally active inhibitor of VEGFR2, was homogeneously suspended in 0.9% saline and administered by oral gavage. Mice were analyzed 12 weeks (pilot study) or 8 weeks after the surgery. To assess the severity of OA induced by surgery, histopathological grading was performed in a blinded manner using the standard Osteoarthritis Research Society International (OARSI) scoring system^42^.

### Histology

Mice limbs and dorsal root ganglia (DRGs) were dissected, soft tissues were removed and fixed in 4% w/v formaldehyde for 48 hours at 4 °C and limbs were decalcified with 0.5 M EDTA (pH 8.0) for 7 to 14 days at 4 °C on a shaker. Complete decalcification was confirmed by digital radiography (*In Vivo* MS FX PRO, Bruker CO). After dehydration using alcohol gradients and infiltration with xylene and paraffin, samples were embedded in paraffin. Paraffin-embedded joints were sectioned at 6–8 µm in a coronal or sagittal plane. A standard protocol was used for Safranin O-fast green staining. Tartrate-resistant Acid Phosphatase (TRAP) staining of sections to identify osteoclasts were performed using a Leukocyte Acid Phosphatase kit (387A-1KT, Sigma-Aldrich). For frozen sections, tissues were cryoprotected in 15% w/v sucrose/PBS for 1hr, then in 30% w/v sucrose/PBS overnight at 4 °C and placed in 30% w/v sucrose/PBS: O.C.T. (1:1) solution for 1 hour. Samples were embedded in Tissue-Tek O.C.T. compound (Sakura, 4583) and transferred to dry ice to solidify the compound. Embedded samples were cryosectioned at 14 µm using a cryostat. Images were acquired with a Nikon Digital Sight DS-Fi1 color camera controlled with NIS-Element acquisition software. Brightness and contrast were adjusted in displayed images using NIS-element acquisition software.

### Immunofluorescence

Paraffin sections were deparaffinized, rehydrated and pre-washed prior to antigen retrieval. For collagen I, collagen ll, collagen X or VEGF immunofluorescence, tissue sections were digested with 2.5 mg per ml of hyaluronidase (H3506, Sigma-Aldrich) in TBS (pH 7.6) for 30 to 60 minutes at 37 °C. After enzyme pre-treatments and blocking, the sections were incubated overnight with mouse monoclonal antibody against collagen II (MS-235-P0, Thermo Scientific) at 1:100 dilution, rabbit polyclonal antibody against collagen X (234196, Calbiochem) at 1:100 dilution, rabbit polyclonal antibody against collagen I (ab21286, abcam) or rabbit polyclonal antibody against VEGF (sc-152, Santa-Cruz) at 1:50 dilution. For CD31 and endomucin immunofluorescence, antigen retrieval was performed in citrate buffer (pH 6.0) for 20 minutes at 95 °C followed by 20 minutes at room temperature. After antigen retrieval, the sections were incubated overnight with rabbit polyclonal antibody against CD31 (Ab28364, abcam) at 1:20 dilution or rat monoclonal antibody against endomucin (sc-65495, Santa Cruz Biotechnology, Inc.) at 1:50 dilution.

For Indian hedgehog (Ihh), hypoxia inducible factor 1α (HIF-1α) and matrix metallopeptidase 13 (MMP13) immunofluorescence, antigen retrieval was performed using 20ug/ml proteinase K in 10 mM Tris/HCl solution (pH 7.5) for 30 minutes at 37 °C. After antigen retrieval, sections were incubated overnight with rabbit polyclonal antibody against Ihh (Ab39634, abcam) at 1:50 dilution, with rabbit polyclonal antibody against HIF-1α (H-206, Santa Cruz Biotechnology) at 1:50 dilution or with rabbit polyclonal antibody against MMP13 (ab39012, Abcam) at 1:100 dilution. After washing with TBS 3 times, sections were incubated with Alexa Fluor 488-labeled goat anti-mouse IgG (A-11001, Life technologies), Alexa Fluor 488-labeled goat anti-rabbit IgG (A-11008, Life technologies), Alexa Fluor 555-labeled goat anti-rabbit IgG (A-21428, Life Technologies) or 488-labeled goat anti-rat IgG (A-11006, Life Technologies) at 1:200 dilution for 60 minutes. After washes with TBS 3 times, the sections were placed in mounting medium with DAPI (H-1500, Vector Laboratories, Inc.) for nuclear staining.

For detection of phosphorylated VEGFR2 (pVEGFR2) in paraffin sections, antigen retrieval was performed in 20 µg/mL proteinase K in PBS for 30 minutes at 37 °C. After antigen retrieval, the sections were incubated overnight with rabbit polyclonal antibody against pVEGFR2 (orb99143, Biorbyt) at 1:100 dilution. After washing with PBS 3 times, sections were incubated with Alexa Fluor 546 goat anti-rabbit IgG (A-11035, Invitrogen) at 1:100 dilution for 60 minutes. After washes with PBS 3 times, the sections were placed in mounting medium with DAPI (H-1200, Vector Laboratories, Inc.) for nuclear staining.

For pVEGFR1, pVEGFR2 and neuronal nuclear antigen (NeuN) immunofluorescence staining of DRG sections, antigen retrieval was performed in 10 mM citrate buffer (pH 6) at 98 °C for 3 minutes. After antigen retrieval, the sections were incubated overnight with rabbit polyclonal antibody against pVEGFR1 (orb191606, Biorbyt) at 1:100 dilution, rabbit polyclonal antibody against pVEGFR2 (orb99143, Biorbyt) at 1:100 dilution, and mouse monoclonal antibody against NeuN (MAB377, Millipore) at 1:500 dilution. After washing with PBS 3 times, sections were incubated with Alexa Fluor 546 goat anti-rabbit IgG (A-11035, Invitrogen) at 1:100 and goat anti-mouse Alexa Fluor 488 IgG (A-11029, Invitrogen) at 1:100 dilution. Fluorescent images were obtained with a Nikon 80i Upright microscope, a Nikon Ti w/Spinning Disk Confocal microscope or Nikon Eclipse Ni upright microscope and associated software. Images of some optic fields were taken using blue and red fluorescence filters, and merged with MetaMorph Software (Molecular Devices LLC).

For detection of NGF and TrkA in paraffin sections of articular cartilage and synovial tissue obtained from knee joints of mice three days after they were given intra-articular injections of VEGF or PBS, sections were stained with rabbit polyclonal anti-NGF (ab6199, Abcam) or rabbit monoclonal anti-TrkA (ab76291, Abcam), followed by incubation with goat anti-rabbit Alexa Fluor 488 IgG (A-11008, Invitrogen).

### TUNEL staining

For TUNEL staining, sections were deparaffinized, rehydrated and pre-washed prior to antigen retrieval. Antigen retrieval was performed using 20 ug/mL proteinase K in 10 mM Tris/HCl solution (pH 7.5) for 30 minutes at 37 °C. TUNEL staining was performed using the *In Situ* Cell Death Detection Kit (11684795910, Roche), according to the manufacturer’s instructions. The sections were incubated with DAPI (H-1500, Vector Laboratories, Inc.) for nuclear staining.

### Micro-CT analysis

Bones were fixed in 4% paraformaldehyde solution for 48 hours. Bones were exchanged into 70% ethanol, stored at 4 °C. The microarchitecture of the proximal trabecular bone and midshaft cortical bone of the tibia was analyzed by μCT (μCT-35, Scanco Medical AG). All scans were analyzed using the manufacturer’s software (Scanco Medical AG) at the Harvard School of Dental Medicine Micro CT Core facility.

### Primary mouse epiphyseal chondrocyte culture

Chondrocytes were isolated from 5-day old *Col2-Cre;Vegf* ^*fl/fl*^ (*CKO* ^*Col2*^) and *Vegf* ^*fl/fl*^ mice as described^43^. Briefly, cartilage was dissected from the femoral heads, femoral condyles, and tibial plateaus of mice and sequentially digested in collagenase D solution (Roche) at 3 mg/mL and then 0.5 mg/mL. Cells were counted and seeded directly into culture chambers at densities of 1.5 × 10^4^ cells and in 12-well plates at a density of 6.25 × 10^4^ cells per well. The cells were maintained as a monolayer in Dulbecco’s Modified Eagle’s Medium (DMEM, 11965-092 Gibco) supplemented with 10% fetal bovine serum (Atlanta Biologicals), L-glutamine (Gibco, 35050-061) and antibiotics (Gibco, 15240-062). Following culture for 4 days in 1% oxygen or 20% oxygen for 4 days, media from 12-well plates were collected for measurements of cytotoxicity by LDH release (Pierce LDH Cytotoxicity Assay Kit, Thermo Scientific #88953), and adhered cells were used for determination of VEGF transcript levels. Adhered cells from culture chambers were examined for apoptosis using Tunel assay (Cell Death Detection Kit Roche), following manufacturer’s instructions.

### Real-time RT-PCR analysis

Total RNA was isolated with an RNeasy mini kit or Trizol reagent (Invitrogen, Carlsbad, Ca) and 500ng were reverse transcribed with Qscript cDNA Super Mix (95048, Quanta Biosciences) to generate single-stranded cDNA. For real-time PCR, cDNA was amplified using iCycler or MyiQ Real-Time PCR Detection System (Bio-Rad Hercules, Ca). Each PCR sample contained iQ SYBR Green Super Mix (170-8880, Bio-rad). Relative mRNA expression was determined using the ^ΔΔ^C_T_ method detailed by the manufacturer (Bio-Rad). Mouse glyceraldehyde-3-phosphate dehydrogenase (Gapdh) or ribosomal 18 s RNA was used for the internal control. All reactions were run in triplicate. Primer sequence information is available upon request.

### VEGF ELISA

VEGF protein levels in cell lysates and culture media were assessed using Quantikine Mouse VEGF Immunoassay (R&D Systems) in accordance with the manufacturer’s protocols; VEGF levels were normalized to protein levels. For measurements of VEGF in culture media, media were collected when cells were 70–80% confluent. The optical density of each well was determined using a microplate reader at 450 nm. The optical density determined with 10% FBS culture medium was used as control.

### Intra-articular VEGF injection

Effects of increased VEGF levels in knee joints were assessed by intra-articular injection of 5 μL phosphate buffered saline (PBS) containing 5 μg human VEGF165 (R&D Systems, 293-VE) or 5 μL PBS only (control) into knees of 24 week-old C57BL/6 J mice (n = 4); joint tissues were harvested for immunohistochemistry 3 days later.

### Statistical analysis

For comparisons of limb lengths, 2-way ANOVA with multiple comparisons were used. For OARSI scoring and immunofluorescence imaging, unpaired two-tailed Student’s t-test were used. For OA progression analysis in *CKO* ^*Flk1*^ mice, a multiple regression adjusted for body weight was used. For longitudinal body weight analysis, 2-way ANOVA with Tukey’s multiple comparison was used. For association between subchondral bone thickening and OARSI grade, a univariate regression model was used. For Real-time PCR analysis, one-way ANOVA with Sidak’s multiple comparison were used. *P* values less than 0.05 were considered significant. Statistical analyses were performed with Stata 13.0 (Stata Corp LP, College Station, TX) and Graph Pad Prism 6 (GraphPad Software, Inc. CA, USA) software.

### Data availability

All relevant data are available from the authors.

## Electronic supplementary material

Supplementary information
