# Supplementary material for: Vascular Endothelial Growth Factor in Cartilage Development and Osteoarthritis
Source: Sci Rep. 2017 Oct 12;7:13027. doi: 10.1038/s41598-017-13417-w (PMC5638804; doi:10.1038/s41598-017-13417-w)

SUPPLEMENTARY INFORMATION

TITLE: Vascular Endothelial Growth Factor in Cartilage Development and Osteoarthritis

AUTHORS:

Masashi Nagao, M.D., Ph.D., John L Hamilton, M.S., Ranjan Kc, Ph.D. , Agnes D Berendsen, Ph.D. , Xuchen Duan, Ph.D. , Chan Wook Cheong, D.M.D, D.M.Sc, Xin Li, M.D., Ph.D., Hee-Jeong Im, Ph.D., Bjorn R. Olsen, M.D., Ph.D.

Supplementary Fig. 1. Body weights of *Col2-CreER;Vegf*$^{fl/fl}$ mice.

Corn oil (Ctrl) or tamoxifen (TAM) were administered to *Col2-CreER;Vegf* $^{fl/fl}$ mice at 2 weeks of age. Body weights in male (**A**) and female (**B**) mice from 3 to 8 weeks were similar in mutants and controls. Lengths of male (**C**) and female (**D**) mice at 8 weeks were similar in the mutant and control femur, but slightly different in the tibia (n=4-5). *P< 0.05. Data represent mean ±SD. 2-way ANOVA with Sidak's multiple comparison tests (**A-D**) were used.

Supplementary Fig. 2. Chondrocytes in normoxic and hypoxic culture conditions.

Levels of LDH release in culture supernatants of chondrocytes isolated from *Vegf*$^{fl/fl}$ (A) and *Col2-CreER; Vegf*$^{fl/fl}$ (B) mice (n=3). Levels are increased in hypoxia (1%) compared to normoxia (20%). No differences between tamoxifen-treated (reduced VEGF) or tamoxifen-untreated (control) cultures are seen. $*P< 0.05$; $**P< 0.01$. Data represent mean ±SD. Unpaired Student's t test was used.

Supplementary Fig. 3. Targeting cells in *Flk1-Cre* and *Col2-Cre* mice.

Sections of tibial epiphysis in *Flk1-Cre;TdTomato* (**A**) and *Col2-Cre;TdTomato* (**B**) mice were stained with antibodies against endomucin (Emcn, green); red represents Tomato-expressing cells; green represents Emcn; blue represents DAPI; scale bars, 250μm.

Supplementary Fig. 4. Body weights and limb lengths in *Flk1-Cre;Vegf*$^{fl/fl}$ mice.

Body weights in male (**A**) and female (**B**) *Flk1-Cre* and *Flk1-Cre; Vegf* $^{fl/fl}$ (*CKO*^{Flk1}) mice from 4 to 8 weeks old (*n*=4-10). Hind limb lengths in males (**C**) and females (**D**) at 4 and 8 weeks of age (*n*=4-8). *P< 0.05. Data represent mean ±SD. 2-way ANOVA with Sidak's multiple comparison tests (**A-D**) were used.

Supplementary Fig. 5. Growth plate and articular cartilage in *Flk1-Cre;Vegf fl/fl* mice.

Sections of tibial growth plate (**A**) and knee joint (**B**) in *Flk1-Cre* and *Flk1-Cre;Vegf* $^{fl/fl}$ (*CKO*^{Flk1}) mice at 9 weeks of age stained with Safranin O-fast green. Scale bars, 250μm.

Supplementary Fig. 6. Body weights before and 12 weeks after DMM surgery.

Body weights of 8 and 20 weeks old male (**A**) and female (**B**) *Col2-CreER;Vegf* $^{fl/+}$ mice and male (**C**) and female (**D**) *Col2-CreER;Vegf* $^{fl/fl}$ (CKO^{Col2ER}) mice administered corn oil (Ctrl) or tamoxifen (TAM) (*n*=3-5). Body weights of 8 and 20 weeks old male (**E**) and female (**F**) *Flk-Cre* or *Flk1-Cre;Vegf* $^{fl/fl}$ (CKO^{Flk1}) mice, *n*=3-5. **P*< 0.05. Data represent mean ±SD. 2-way ANOVA with Sidak's multiple comparison tests were used (**A-F**).

Supplementary Table 1. Number of pups from different genotype combinations

Male	Female	Total N of pups	N of CKO^{Col2} mice (expected)
Vegf $^{fl/fl}$	*Col2-Cre;Vegf* $^{fl/+}$	69	14 (17)
Vegf $^{fl/+}$	*Col2-Cre;Vegf* $^{fl/+}$	23	3 (2.9)
Col2-Cre;vegf $^{fl/+}$	*Vegf* $^{fl/fl}$	13	1 (3.3)
Col2-Cre;vegf $^{fl/+}$	*Vegf* $^{fl/+}$	121	0 (15.1)

Supplementary Table 2. Bone parameters in *Col2-Cre* and *Col2-Cre;Vegf* $^{fl/+}$ mice.

	Male			Female		
	Col2-Cre<br>(95% CI)	***Col2-Cre;Vegf*** $^{fl/+}$<br>(95%CI)	*P* value	**Col2-Cre**<br>(95%CI)	***Col2-Cre;Vegf*** $^{fl/+}$<br>(95%CI)	*P* value
**BV/TV**	0.14<br>(0.11-0.17)	0.09<br>(0.06-0.12)	*0.03	0.07<br>(0.06-0.09)	0.055<br>(0.035-0.074)	0.14
**Tb.N**	4.63<br>(4.31-4.95)	4.08<br>(2.64-5.53)	0.10	3.00<br>(2.49-3.52)	2.40<br>(1.89-2.91)	0.14
**Tb.Th**	0.043<br>(0.037-0.050)	0.040<br>(0.032-0.049)	0.52	0.038<br>(0.034-0.041)	0.040<br>(0.038-0.042)	0.32
**Tb.Sp**	0.21<br>(0.19-0.23)	0.25<br>(0.16-0.35)	0.07	0.35<br>(0.29-0.42)	0.43<br>(0.34-0.51)	0.16
**C. Th**	0.21<br>(0.20-0.22)	0.22<br>(0.19-0.24)	0.46	0.19<br>(0.18-0.21)	0.20<br>(0.18-0.21)	0.77

Quantification of Trabecular Bone Volume /Tissue Volume (BV/TV), Trabecular Number (Tb.N), Trabecular Spacing (Tb.Sp), Trabecular Thickness (Tb.Th) and Cortical Thickness (C.Th) in tibia of *Col2-Cre* and *Col2-Cre;vegf* $^{fl/+}$ mice (n=4). 95% confidence interval, 95%CI, *P< 0.05. Unpaired student's t-test was used.

Supplementary Table 3. Association between genotype and subchondral bone thickening 12 weeks after DMM surgery

	Coefficient (95% CI)	*P* value
Col2-Cre;Vegf $^{fl/+}$	1.33 (-0.35 to 3.01)	0.09
Col2-Cre	1.45 (1.10 to 1.82)	<0.01*
CKO ^{Col2}	1.51 (0.45 to 2.56)	0.01*
CKO ^{Col2} ***with no tamoxifen***	1.15 (0.22 to 2.07)	0.02*
CKO ^{Flk1}	1.00 (0.03 to 1.97)	0.04*
Flk1-Cre	1.00 (0.50 to 1.49)	<0.01*

**P*< 0.05. Univariate regression analyses were used. Dependent variables were the OARSI grade of subchondral bone thickening and independent variables were genotypes.

Supplementary figure 1

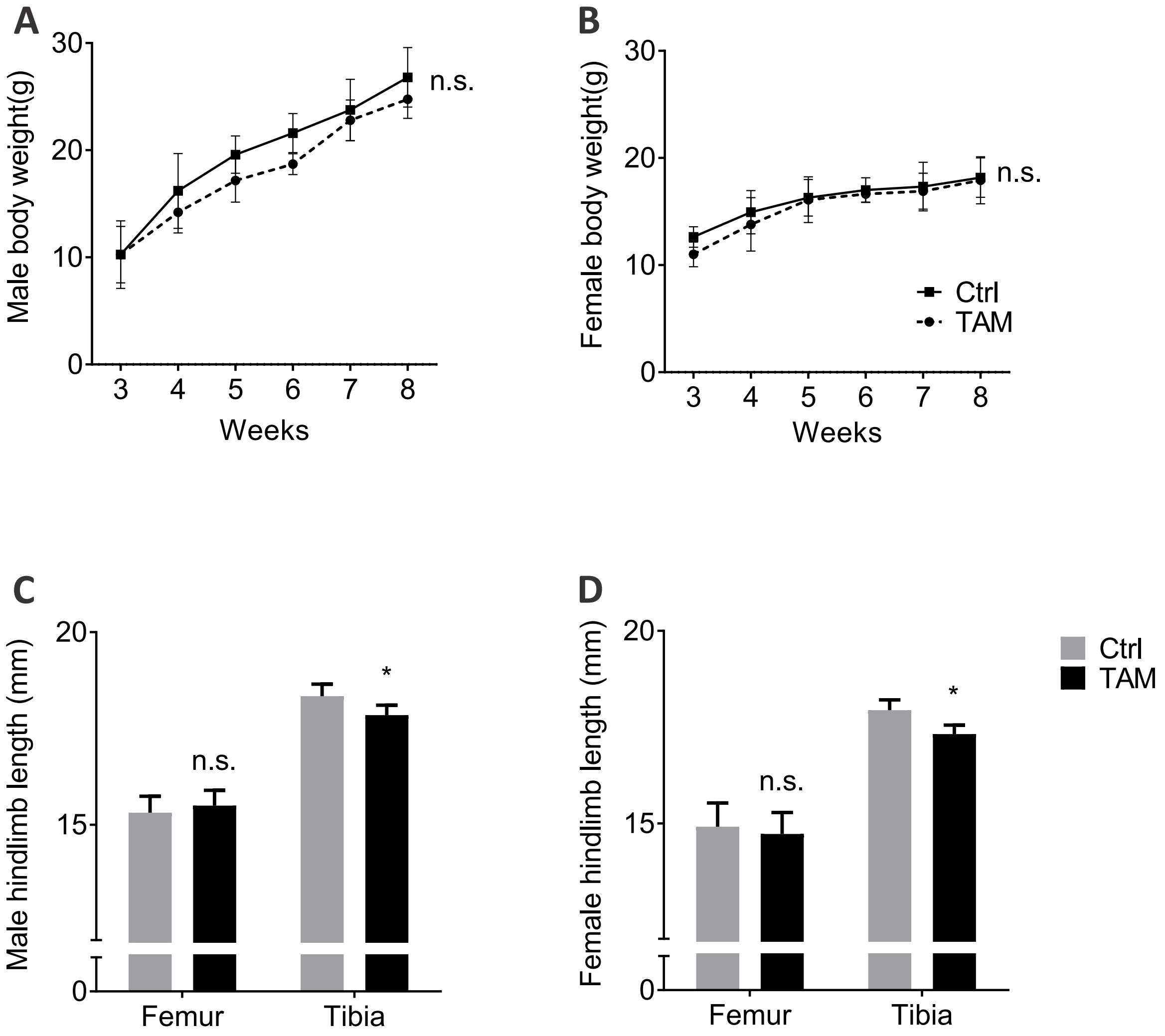

Supplementary figure2

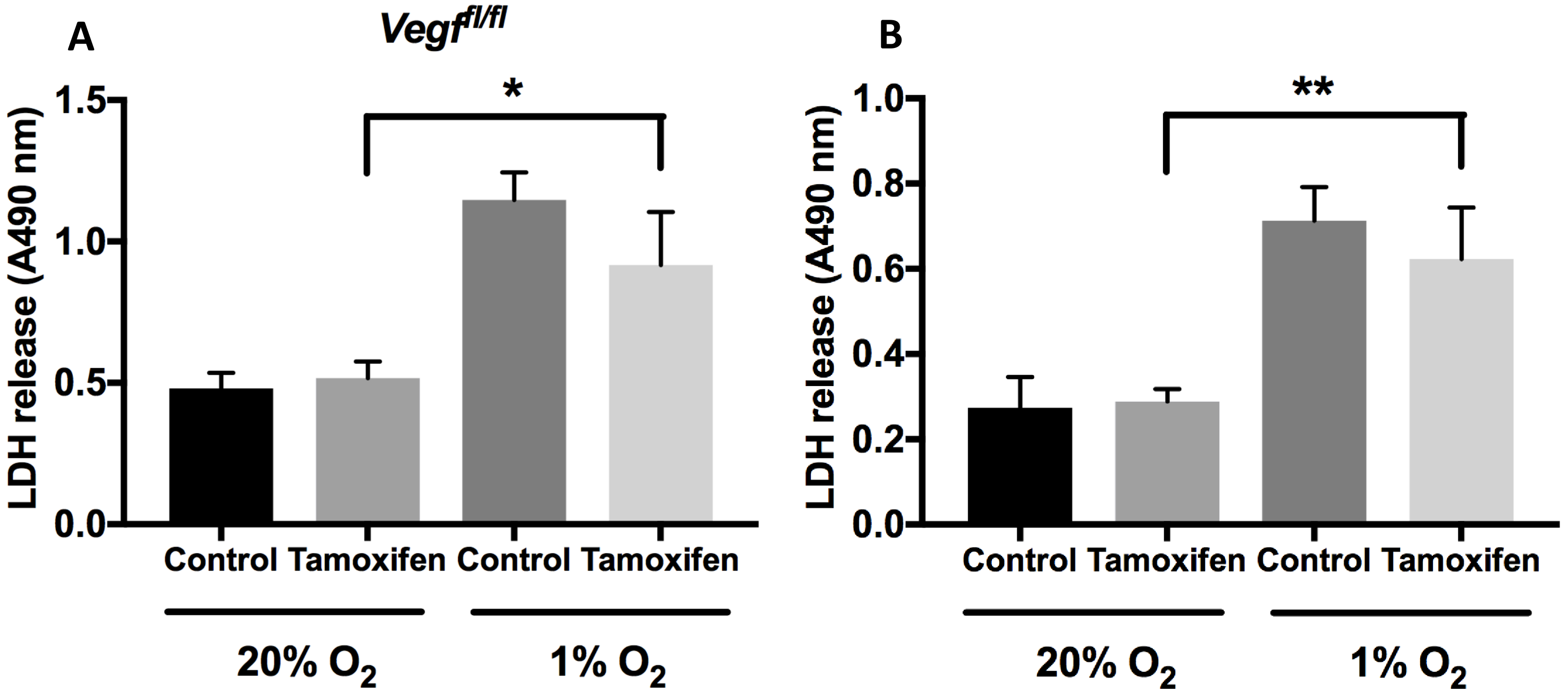

Supplementary figure 3

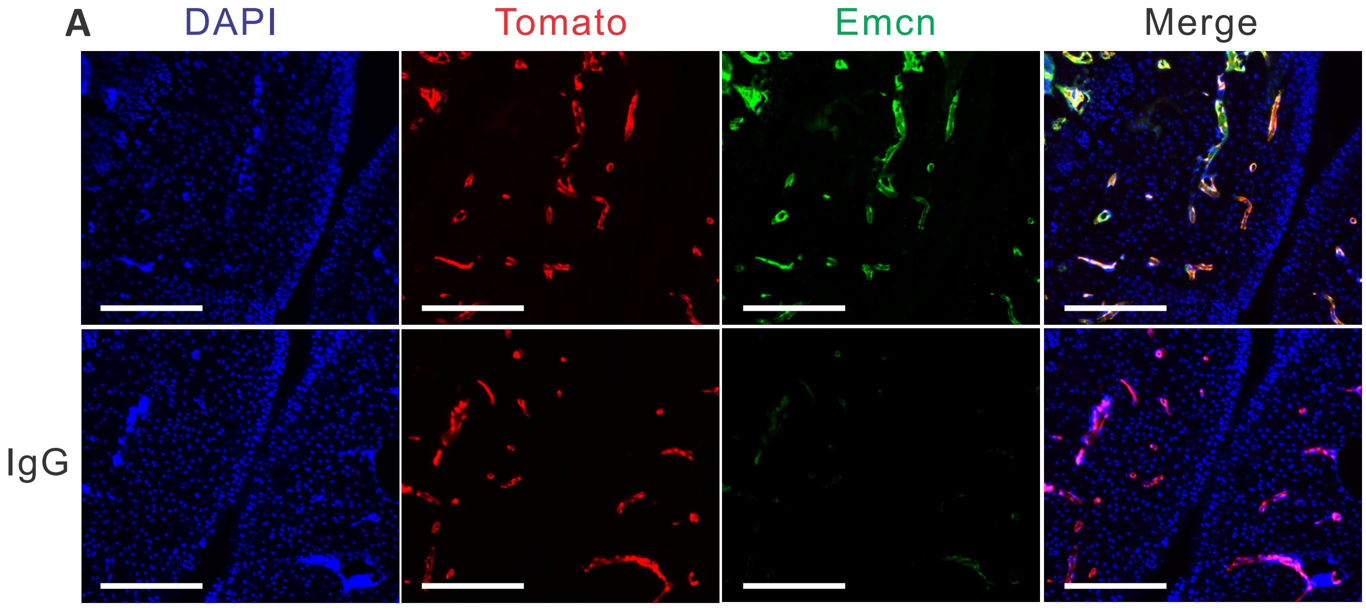

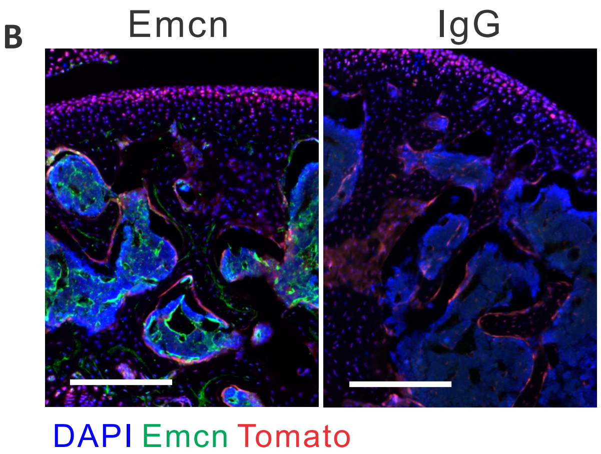

Supplementary figure 4

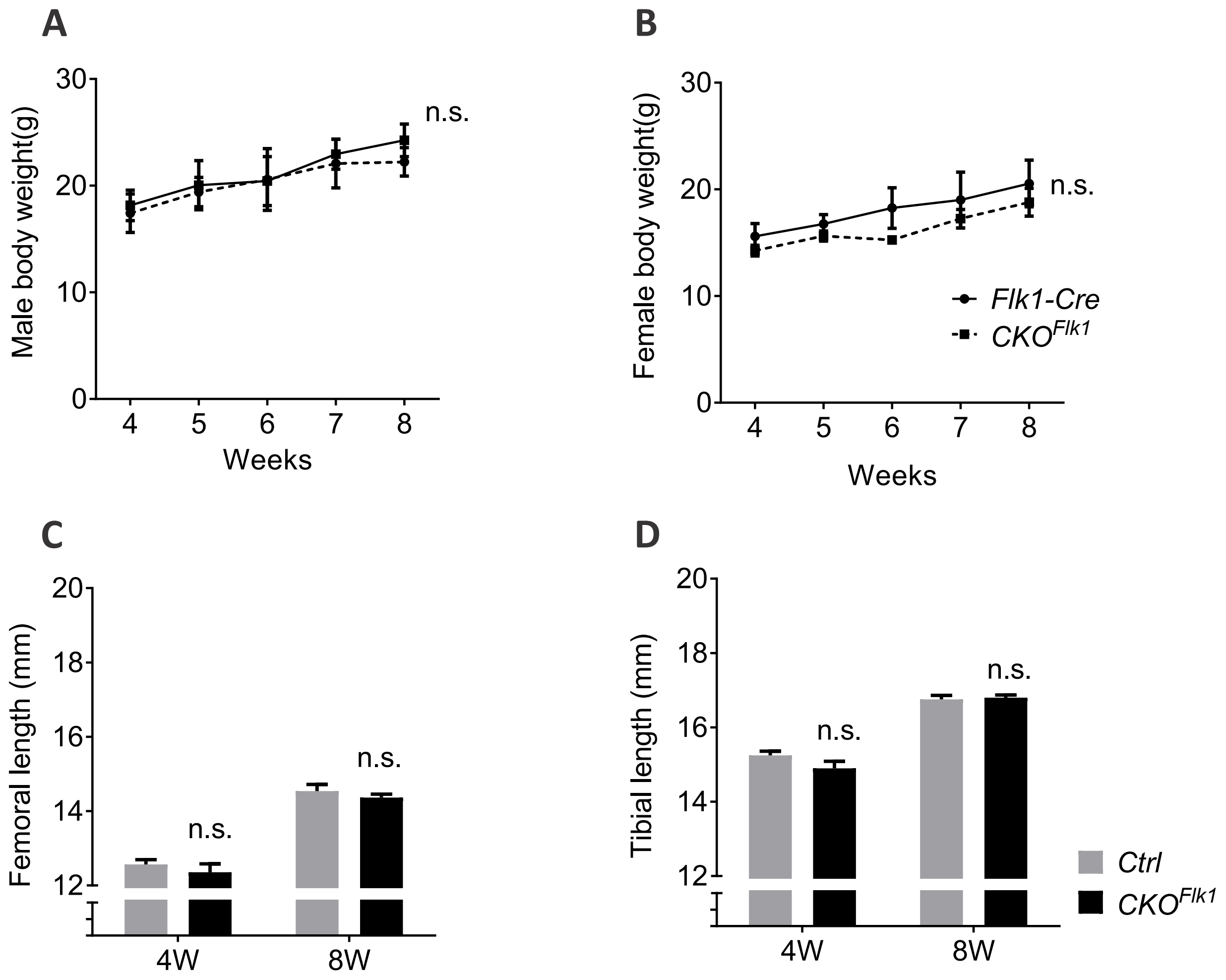

Supplementary figure 5

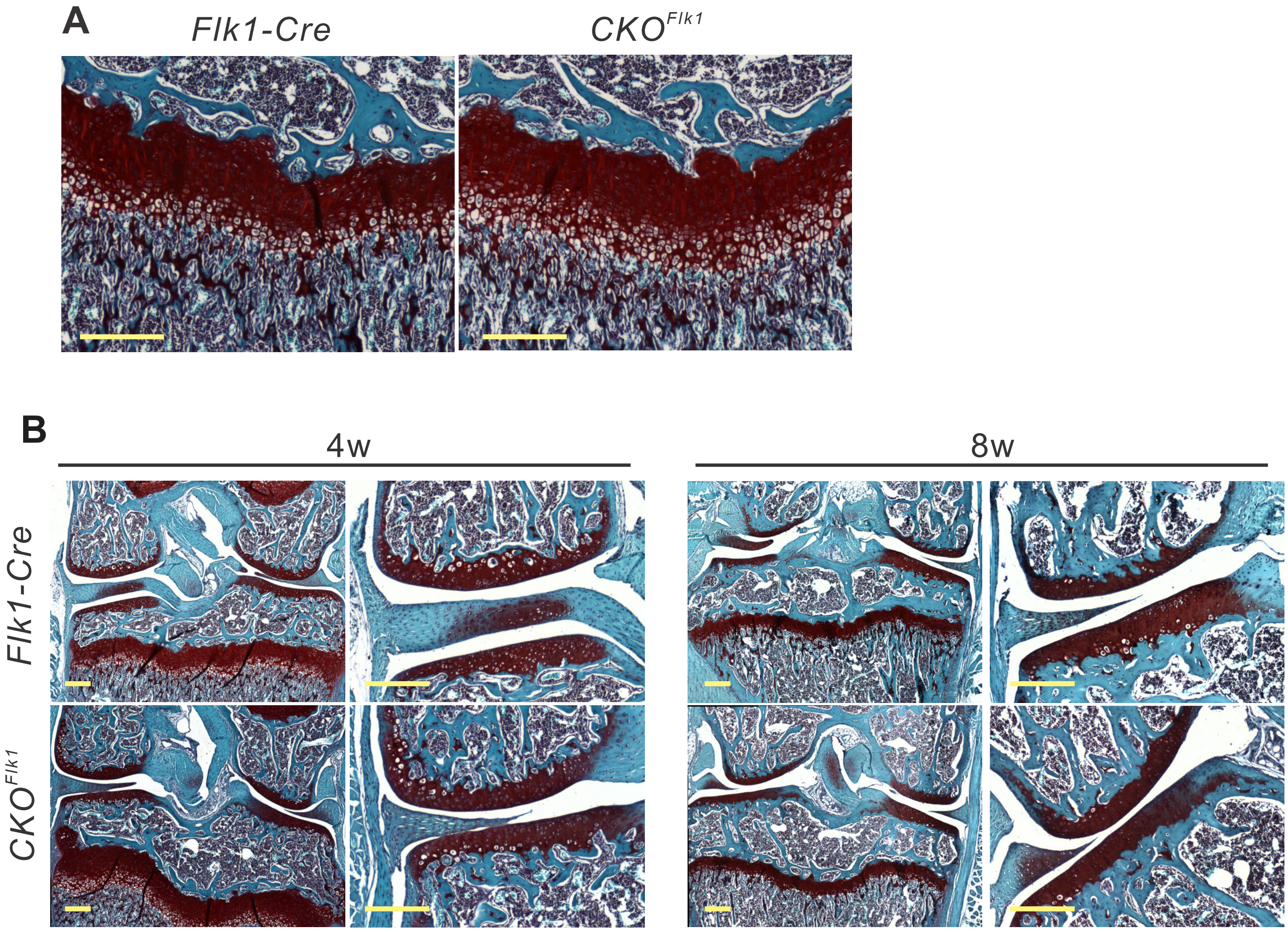

Supplementary figure 6

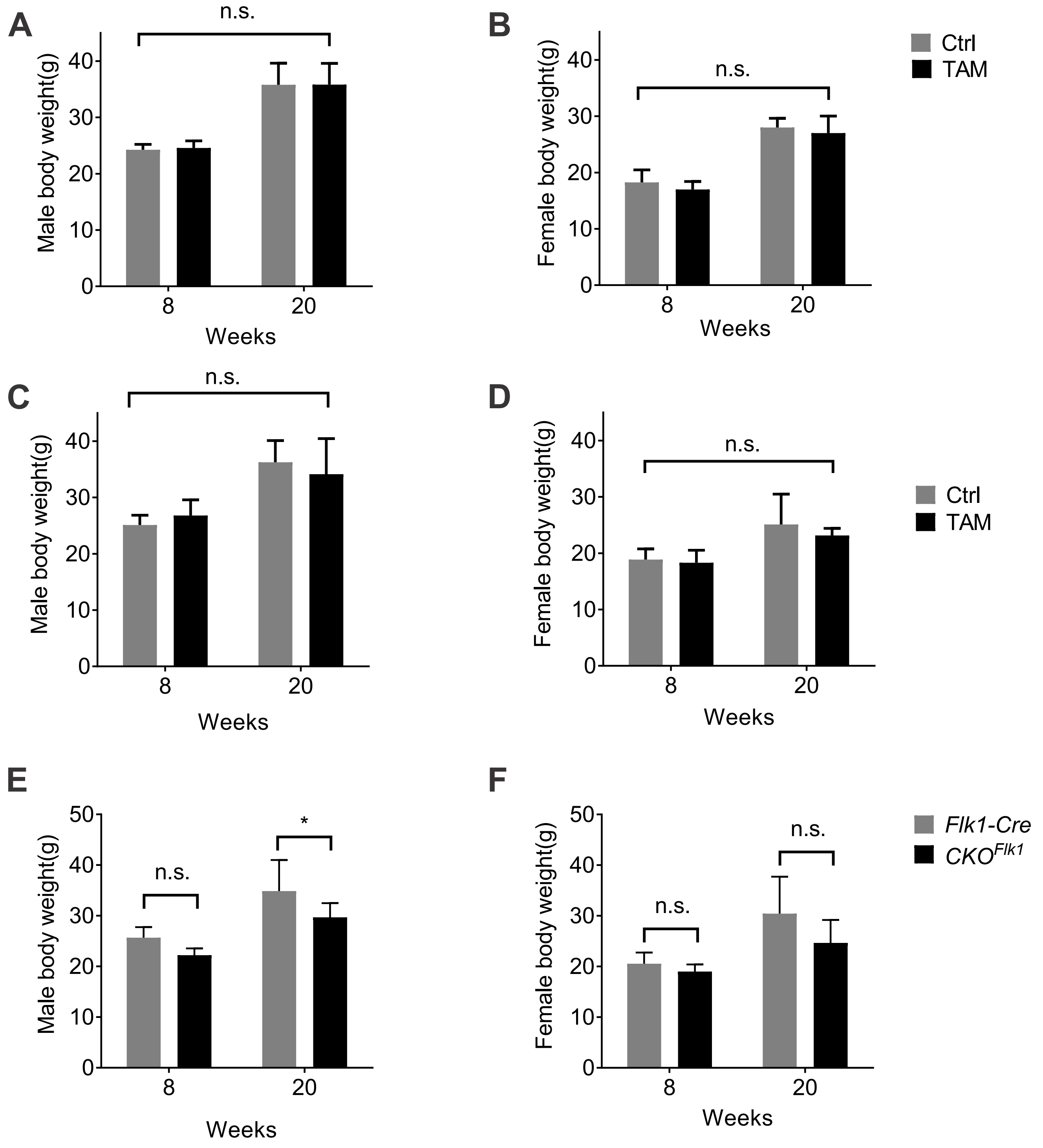

Supplement: Supplementary file 1 — Supplementary information [file 41598_2017_13417_MOESM1_ESM.pdf]
